# Using Geodesign as a boundary management process for planning nature-based solutions in river landscapes

**DOI:** 10.1007/s13280-020-01435-4

**Published:** 2020-12-17

**Authors:** Sarah Gottwald, Jana Brenner, Ron Janssen, Christian Albert

**Affiliations:** 1 Institute for Environmental Planning, Leibniz University Hannover, Hannover, Germany; 2 Department of Spatial Economics, Vrije Universiteit of Amsterdam, Amsterdam, The Netherlands; 3 Institute for Geography, Ruhr-University, Bochum, Germany; 4 Present Address: Department of Geography, University of Hildesheim, Hildesheim, Germany; 5 Institute of Geography, Chair for Environmental Analysis and Planning in Metropolitan Regions, Ruhr University Bochum, Bochum, Germany

**Keywords:** Freshwater, Land use change, Participatory mapping, Planning support tool, River management, Touch table

## Abstract

Planning with nature-based solutions (NBS) presents a participatory approach that harnesses actions supported by nature to address societal challenges. Whilst Geodesign may facilitate participatory planning, manage boundaries between participants, and assess impacts of NBS, empirical insights remain scarce. This paper aims to develop and test a Geodesign process for planning with NBS, and to evaluate its contributions to boundary management. In a one-day Geodesign process, eleven stakeholders delineated priority areas, changed land uses, and observed resulting impacts on ecosystem services. Contributions to boundary management were evaluated regarding translation, communication and mediation functions, as well as perceived attributions of credibility, salience, and legitimacy. Results include spatial NBS scenarios and insights into contributions to boundary management: translating scenario stories into maps differed depending on the stakeholders involved; communication can be easily facilitated; yet mediation using an indicator tool led to frustration. Geodesign can indeed facilitate NBS co-design but needs to be integrated into a larger collaborative process.

**Electronic supplementary material:**

The online version of this article (10.1007/s13280-020-01435-4) contains supplementary material, which is available to authorized users.

## Introduction

Planning with nature-based solutions (NBS) responds to calls for changes in river management: (1) there is a shift from grey, or technical, infrastructure towards more nature-based, or green, infrastructure and solutions (Fliervoet et al. 2013; Albert et al. 2019); and (2) local citizens and their values are more deeply integrated with the management process through participatory processes and innovative governance models (Fliervoet et al. 2013; Westerink et al. 2017). NBS is a recently proposed concept in both practice and science that provides solutions to societal challenges inspired and supported by nature by bringing together established ecosystem-based approaches such as ‘ecosystem services’, ‘green-blue infrastructure’, ‘ecological engineering’, ‘ecosystem-based management’, and ‘natural capital’ (Nesshöver et al. 2016; Editorial 2017). NBS are actions that ‘(i) alleviate a well-defined societal challenge, (ii) utilise ecosystem processes of spatial, blue and green infrastructure networks, and (iii) are embedded within viable governance or business models for implementation’ (Albert et al. 2019, p. 15). Examples for NBS in river basins are revitalising flood plains, removing dams, planting forests, or restoring rivers (NWRM 2015; Guerrero et al. 2018; Lafortezza et al. 2018). It has been shown that the protection of upstream forests supports downstream flood protection and led to a reduction in flood damage costs in a German case study (Barth and Döll 2016).

Although systematically integrating diverse stakeholders has been identified as a key requirement for successfully planning and implementing NBS (European Commission 2015), the systematic integration in practice remains challenging and under-explored (Raymond et al. 2017). NBS need to be adapted in the context of social-ecological systems, where people are crucial agents for change. Their backgrounds, abilities, cultural setting, and power relations influence their understanding of, and interest in, land use changes (Stedman 2016). Further, Tengö et al. (2014) argue that knowledge differs according to its original system (local, indigenous, scientific); e.g. local knowledge is often developed through experiential processes over long time periods. Joining multiple stakeholder perspectives into a multi-knowledge evidence base arguably enriches the understanding of the overall picture. However, this process is not straightforward and can be complicated by boundaries existing between different social groups with diverging interests and frames of knowledge (e.g. scientific knowledge as opposed to local knowledge), and between different institutional approaches (Westerink et al. 2017; Henze et al. 2018).

It follows that planning with NBS should recognise and sensibly manage the boundaries between stakeholders. Instead of being seen as obstacles or limitations, boundaries can also be understood as a common dynamic interface upon which stakeholders collaborate to solve planning and management challenges. This collaboration can be understood as boundary management, that is, a process which seeks to overcome boundaries between stakeholders. It can be based on three key principles: (1) enabling meaningful participation of relevant stakeholders, (2) generating the setting for liable processes and results, and (3) producing boundary objects (Cash et al. 2003; Clark et al. 2016). Boundary objects are information co-produced by various stakeholders (Cash et al. 2003). Star (2010) stresses that boundaries can be understood as a shared space, and that objects are something people interact with, or towards, thus imbuing them with a dynamic nature. Boundary objects allow for collaboration without consensus (Star 2010) because they provide materiality within a shared space. Boundary objects are characterised by “interpretive flexibility”, that is, they are “both plastic enough to adapt to local needs and constraints of the several parties employing them, yet robust enough to maintain a common identity” (Star and Griesemer 1989, p. 393). Their creation is usually not arbitrary but motivated or forced by common information and work requirements (Star and Griesemer 1989; Star 2010). Accordingly, Cash et al. (2003) propose that boundary management could help to balance trade-offs between key attributes of effective scientific information, specifically perceived scientific credibility, practical salience, and procedural legitimacy (CSL). In its operationalisation, boundary management is characterised by three functions: translation, communication, and mediation (Cash et al. 2003; Clark et al. 2016).

One promising approach that aims to facilitate boundary management is Geodesign. Geodesign is a planning-support process and can be defined as ‘a design and planning method which tightly couples the creation of design proposals with impact simulations informed by geographic contexts, systems thinking and digital technology’ (Steinitz 2012, p. 12). Geodesign tools such as mapping and sketching (Ervin 2011; Raumer and Stokman 2013; Janssen et al. 2015) offer opportunities to transform ideas into specific spatial information, thereby stimulating communication. Analysis tools, including multi-criteria analysis or impact simulation models (Eikelboom and Janssen 2015a), can potentially mediate between diverse opinions by providing facts and figures. Wissen Hayek et al. (2016) argue that Geodesign may facilitate boundary management between science and practice as it translates information and, therefore, promotes common understanding and conflict mediation. Beyond this, Geodesign workshops may produce geographic maps that can serve as boundary objects. Geodesign processes hold special potential for the use of NBS planning because they promote the systematic integration of diverse stakeholders and their knowledge into the planning process. They are suitable for exploring planning alternatives, for example the development of a spatial adaptation strategy. Spatially translating scenarios in the context of Geodesign has resulted in evaluations which differ from scenarios’ evaluations without such translation, specifically in terms of providing less support for ‘business as usual’ or a full NBS scenario, and more support for an intermediate scenario (Gottwald et al. 2020). Whilst Geodesign could thus arguably provide useful tools and methods with which to facilitate participatory planning, manage boundaries between participants, and assess the impacts of NBS, empirical insights on its actual capacities remain scarce.

This paper aims to develop and test a Geodesign process for planning with NBS, and to evaluate its outputs and contributions to boundary management. Our analysis is guided by the following research questions:

1. Which boundary objects can be co-developed in a Geodesign process for planning with NBS, and how do those outputs differ across different tools applied, interfaces used, and stakeholder groups involved?
2. How did the Geodesign process facilitate boundary management amongst the stakeholders involved in river landscape planning?

## Materials and methods

### Geodesign case study

**Fig. 1:**
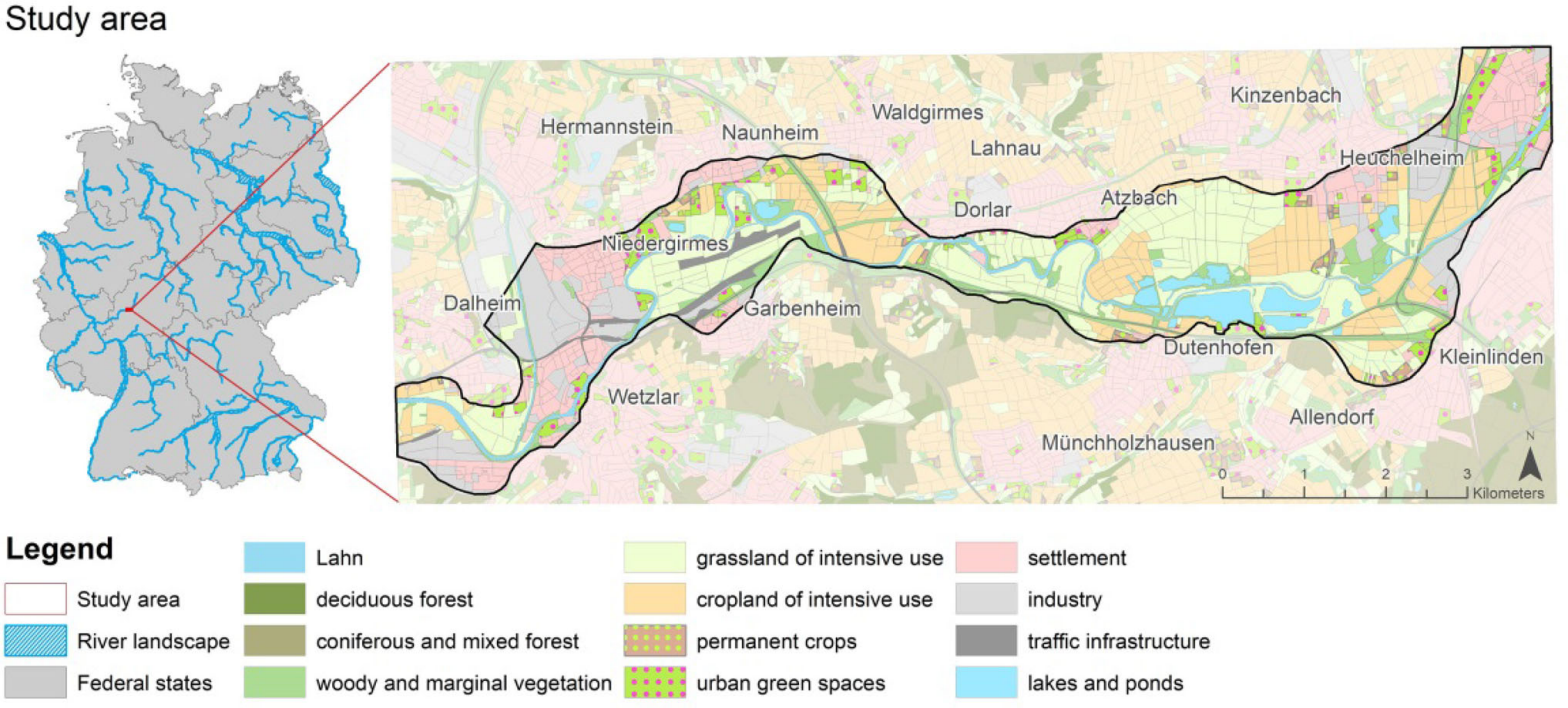
Study area in the overall context of German river landscapes and the current land use in the morphological floodplain. Data source: Digital Basic Landscape Model (ATKIS 2016) provided by Federal Agency for Cartography and Geodesy

A stretch of about 31.6 km of the Lahn river and 2259 ha of the river landscape in Hesse, Germany, was selected as a case study for the Geodesign process (Fig. 1). Like many rivers in Europe, the Lahn was straightened and dammed in the mid-19th century for hydropower generation. Together with a loss of former floodplain areas for settlement and infrastructure, and an intensification of agriculture, the river today illustrates ecological deficits, according to the Water Framework Directive (LiLa 2019). Achieving ecological improvements remains challenging in terms of aligning the legitimate interests of stakeholders, who range from hydro-energy producers to those who pursue recreational boating, fishing, biking and hiking (Albert et al. 2019). Technical infrastructure here requires maintenance, and there has been on-going discussion over changing the river’s status from federal to state waterway—which has implications for the financial and administrative responsibilities of maintaining the technical infrastructure for flood protection and navigability. In combination with the river’s poor-to-moderate ecological status, this situation crucially raises the question of rethinking the current river management strategy to provide natural processes with a higher priority. The study area is part of a transdisciplinary project, and cooperation within this has led to the realisation of the current research. It offers a variety of challenges (e.g. ecological quality, technical infrastructure) which potentially can be solved by NBS.

### Geodesign participants and existing boundaries

As a response to these challenges for the future of the river landscape, the integrated EU life project Living Lahn (LiLa, lila-livinglahn.de) was founded. Workshop participants were directly recruited by the organisers, and eleven stakeholder experts participated in the Geodesign process, representing all institutions involved in the LiLa project: The Hessian Ministry for the Environment, Climate Protection, Agriculture and Consumer Protection (HMUKLV), the Governmental Authority of Gießen (RPGI), the Ministry of Environment, Agriculture, Nutrition, Viniculture and Forestry of Rhineland-Palatinate (MUEEF), the Directorate for Infrastructure and Approval North (SGD Nord), the Waterways and Shipping Office Koblenz (WSA Koblenz), and the German Federal Institute of Hydrology (BfG), (Supplementary Material S1). The collaboration with LiLa presents a unique situation due to the fact that these different institutions came together to work (for 10 years) on a long-term strategy for the sustainable development of the river landscape. Boundaries between stakeholders exist in different dimensions: (1) these institutions work at different spatial levels (national, federal and regional) and thus usually have a different spatial focus; (2) they claim different competencies and responsibilities (e.g. the decision-making competence of the RGGI, the consultancy competence of the BfG); (3) participants have different educational backgrounds; and finally (4) they pursue different interests in terms of themes such as river navigation and nature conservation (Henze et al. 2018). Nevertheless, despite these boundaries LiLa participants identified a number of common interests, such as enhancing ecological quality and recreational opportunities (Henze et al. 2018).

### Digital design and database

**Fig. 2:**
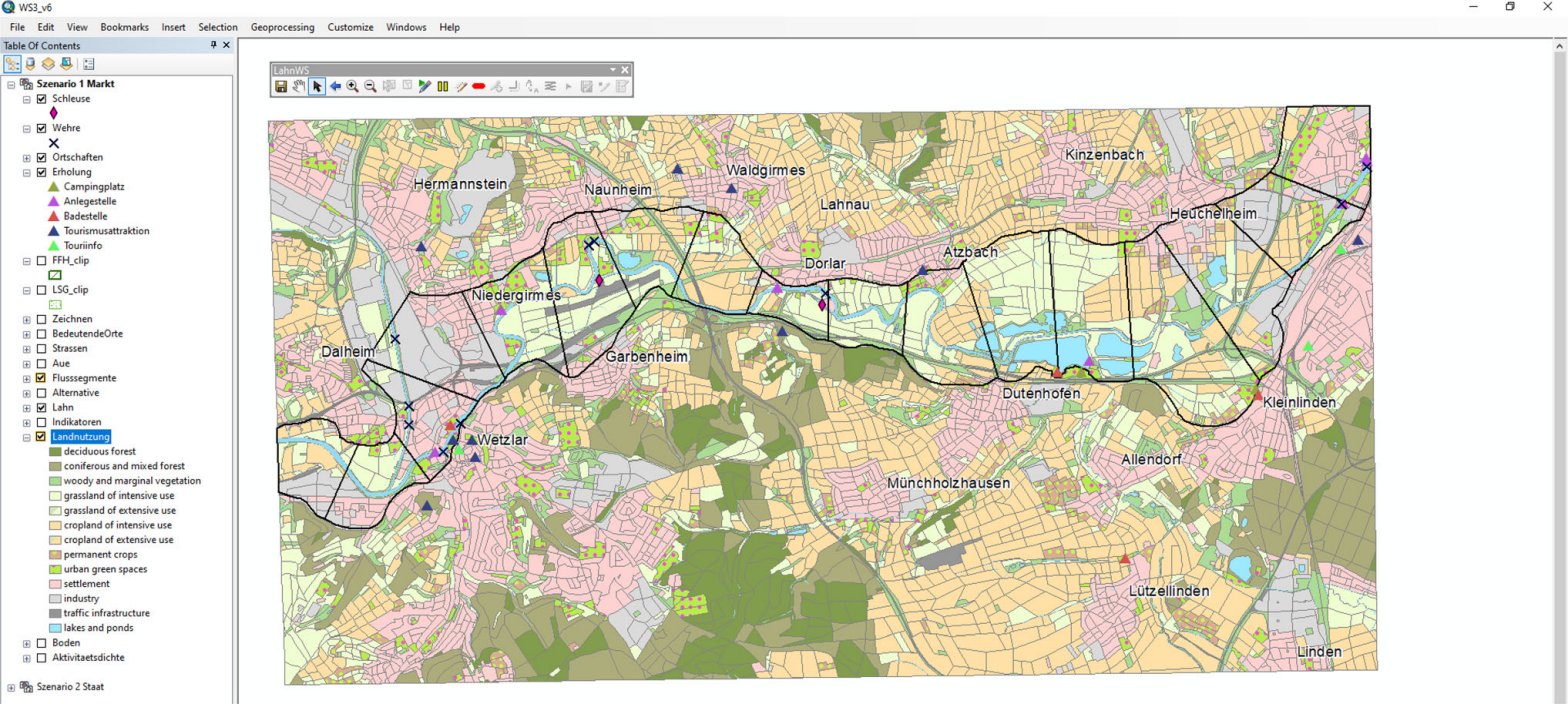
The toolbar was adapted to the needs of the workshop and reduced to a minimum of items (three general tools, three zooming tools, ten tools for drawing and writing and four land use change tools) to create a clear surface respondents could interact with

A digital and interactive workshop design was chosen using two touch tables (Iyama 33 and 52, see Supplementary Material S2) and a specifically prepared GIS interface (ArcGIS 10.6 with the CommunityViz extension, see Fig. 2). The interface enabled co-designing spatial scenarios in a directly geo-referenced way and the simultaneous assessment of likely NBS co-benefits and co-costs, in terms of impacts on ecosystem services provision. In order to allow all participants to actively engage in the spatial co-design, participants were divided into two groups (Group A and Group B), both of which were provided with access to a touch table and identical Geodesign software. Participants were assigned to one of the two groups by the workshop organisers in such a way that both groups included the diversity of institutions and contained at least one person who was involved in developing both the Market and the State scenario, respectively.

Participants were provided with spatial data on land use, protection status, settlements, weirs, sluices and dams, and recreational infrastructure. The data were derived from a ATKIS Base Digital Landscape Model (BKG 2016) provided by the Federal Agency for Cartography and Geodesy. Information on recreational infrastructure had been enhanced using Open Street Maps (camping areas, canoe clubs or ports). The river course of the State scenario (see Box 1) was adjusted according to the storyline (river restoration and removal of locks) using the historical river course of 1818/19 and 1801–1820 (HVBG 2016). Additionally, the study area was divided into river segments of 1 km each, as had been done in earlier assessments of river landscapes (BMUB and BfN 2009). The division into segments was designed to ease communication and to serve as the basis for more local-level impact assessments.

### NBS planning in the Lahn river landscape

NBS are required to address societal challenges, utilise ecosystem processes, and become embedded within viable governance or business models for implementation (Albert et al. 2019). The challenge addressed in this workshop had been previously defined by the participants in their main project aim, which is the ecological enhancement of the river and concomitant wish to make the river landscape more liveable (LiLa 2019). Further, NBS utilise ecosystem processes to provide ecosystem services. So as to incorporate these criteria explicitly, the impact of the proposed land use changes on selected ecosystem services (ES) was calculated on the spot and then presented to participants for further discussion (see detailed description below). Finally, the NBS were designed for two scenarios which differed in their governance and business models of the implementation of NBS. Whilst this third criterion was not explicitly focused on in this workshop, differences in the designs could be observed between a Market and a State scenario (Box 1).

### Conceptual framework of the Geodesign process and its contribution to boundary management

**Fig. 3:**
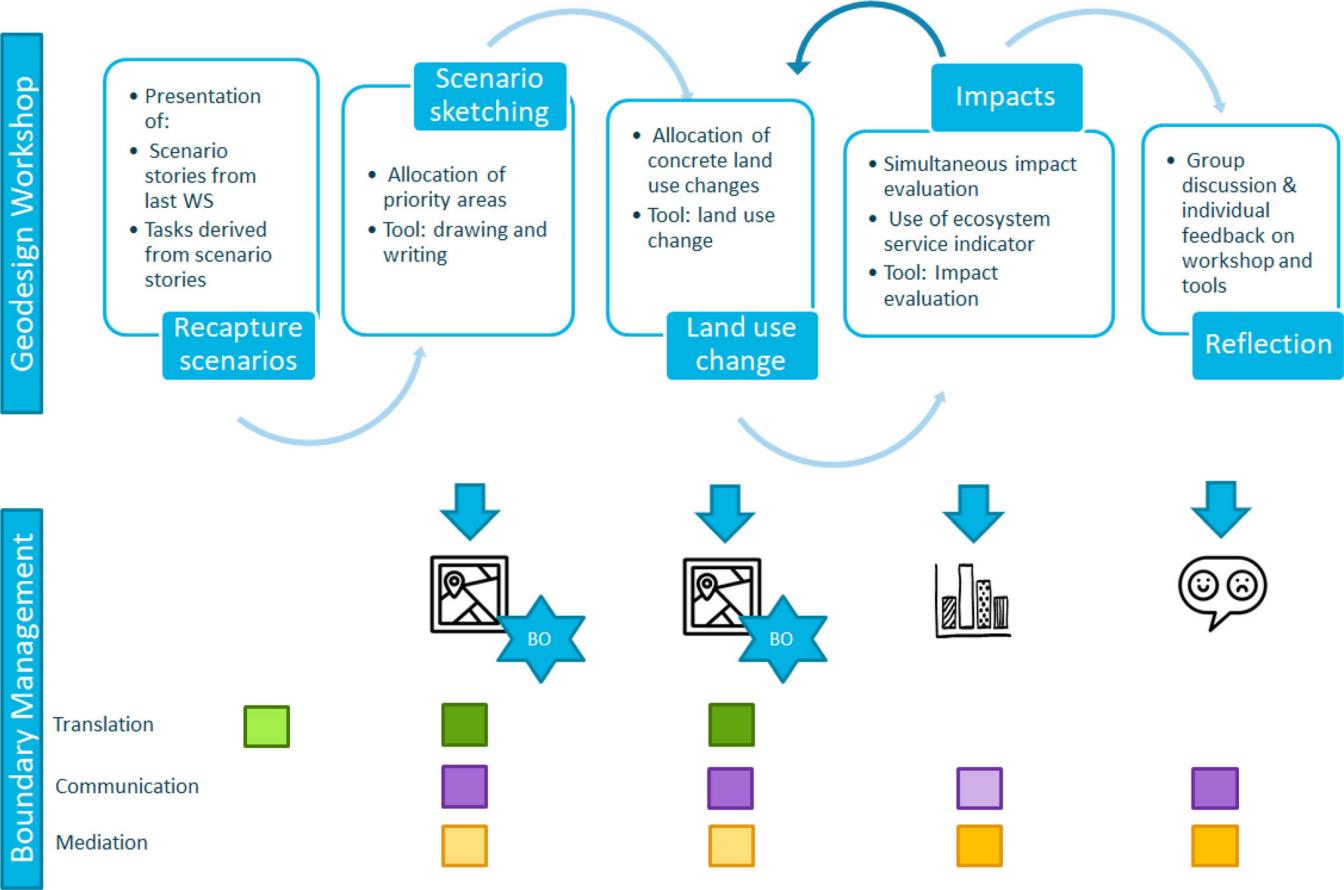
Workshop concept: the upper part illustrates the main steps of the geodesign workshop. The lower part highlights the co-production of boundary objects (BO) the functions of boundary management. A darker shade indicates a strong potential to fulfil the function, less saturated colour indicates a weaker potential of that function

Our conceptual understanding of the Geodesign process and its potential contributions to boundary management is as follows: The Geodesign process was implemented as a workshop consisting of five essential steps (Fig. 3) to co-develop and explore spatial scenario maps in consideration of NBS. The steps included (1) recapitulating scenario stories developed in a prior workshop, (2) scenario sketching, (3) allocating land uses, (4) exploring impacts, and (5) reflecting challenges and opportunities of Geodesign for river landscape planning. Three tools were implemented within the five steps – drawing and writing, land use change, and impact evaluation – which allowed participants to work on the tasks for each of the scenarios. Our assumed contributions to boundary management differed across each step; and we expected Steps 2 and 3 to support all three functions of translation, communication and mediation, whilst expecting the other steps to support only one or two functions (Fig. 3). Several outputs were created, such as maps, impact assessments, and discussion notes.

In a first step, NBS scenario narratives co-produced in the previous workshop were presented and the workshop method was introduced to participants. As the scenarios were translated into specific tasks prior to the workshop, Fig. 3 shows a lightly shaded box for the translation function. In Steps 2 and 3 participants developed spatial scenarios. They translated the scenarios and their specific ideas and perspectives into spatial information; the tool and tasks stimulated communication and a facilitator in each group mediated the process, as illustrated by the three boxes in Fig. 3. As boundary objects these maps support interpretive flexibility because they contain robust information yet can be read from different perspectives. In Step 4, impacts of NBS on an exemplary set of ecosystem services are assessed, and no boundary objects are directly co-produced. However, this significantly influences how boundary objects are developed in Step 3 by highlighting co-benefits and co-costs. Impacts on ES are communicated to the participant and meant to mediate their decision process, and simultaneously stimulate communication between participants.

Box 1: Scenarios In a previous workshop, the same participants engaged in a participatory exercise to develop four scenario stories of how the case study might develop in the future, under more or less consideration of NBS. The workshop presented in this paper selected the two scenario stories which included a strong collaboration with nature and, thus, enabled the design of NBS measures. The two scenarios, ‘Market’ and ‘State’, were further translated into spatial scenario maps. The two scenarios were selected because they aligned well with stakeholder interests and enabled the exploration of NBS in river landscape development.

The Market scenario was guided by the principle of social-environmental entrepreneurship. For this scenario participants assumed that market forces played a strong role in landscape development and that the use of natural resources was a high priority for them. The envisioned future landscape was heterogeneous with rivers and lakes intensively used by local citizens, companies, and tourists for largely recreational purposes. Few industries remained in the area, and new settlements were established in prime locations along the embankment. The main challenges in the scenario were seen to lie in increased flood risks, the financing of projects, strong competition for land, and exacerbating conflicts over land use.

**Table:** 

Proposed scenario sketching tasks	Land use change tasks
1. Please locate on the map areas of especial importance for agriculture, recreation, nature protection	1. A minimum of 30% of the agricultural areas within the floodplain should be in extensive use
2. Please identify locations for camp grounds, canoe stations, swimming areas, etc. and locate them on the map	2. Allocate 40 ha of forest on the floodplain
3. Please identify potential bike and hiking trails and locate them on the map	3. Increase recreational value in at least one land use parcel (from red to orange, or from orange to green)
	4. Increase built-up environment by 20ha without reducing values of the indicator pollination and climate protection
	5. Allocate 10 ha of industrial area
	6. The recreational quality should not decrease due to changes in land use

The State scenario was guided by the principle of cooperation with nature, with the assumption of the government playing a strong role in regulating development. Scenario assumptions included the establishment of large wetlands at the upper region of the river, extensive restoration activities, the removal of several locks, the establishment of extensive agriculture within the floodplain area, good water quality, new forms of living and housing in the flood plain (swimming houses), a high importance of recreation, low industrial activity, and a plethora of nature observation opportunities. Key challenges that were identified included trade-offs between ecosystem processes (e.g. water power and fish protection), increasing climate change, the risk of droughts and floods, neophytes, and too many tourists.

**Table:** 

Proposed scenario sketching tasks	Land use change tasks
1. Please identify on the map areas of especial importance for agricultural use (with temporal flooding), for the expansion of the floodplain, recreation and nature protection	1. Increase the amount of extensive agriculture (cropland and grassland) to 40%
2. Identify and locate on the map measures for ecological connectivity, for example fish ladders	2. Increase indicator values for climate protection and pollination in at least one river segment
3. Allocate paths and entry points to enable access to the river	3. Reduce the area of built-up environment in the morphological floodplain from 320 ha to 250 ha
4. Identify and locate on the map recreational opportunities, such as cycling paths, beaches, etc.	4. Reduce industrial area within the floodplain from 290 ha to 250 ha
	5. Sustain the existing recreational quality despite changes in land use
	6. The recreational quality should not decrease due to changes in land use

### Step 1 Recapitulating scenario stories

The Geodesign workshop started with a 15-minute recapitulation of scenario stories with NBS developed in a previous workshop with the same group of stakeholders (Box 1). Participants received a set of spatial scenario creation tasks that were suggested by the workshop facilitators and represented a translation of the scenario stories. The specific tasks were proposed so as to ease the co-creation of maps from stories, which in practice often presents difficulties (Arciniegas et al. 2013). For example, a scenario story suggesting a forest restoration in the floodplain was translated into: “Allocate *40 ha* of forest in floodplain!” (Box 1, Supplementary Material, S3).

### Step 2: Scenario sketching

In the second step, participants were asked to sketch scenarios on the map by identifying priority areas and important infrastructure (polygon, point or line features). 15 to 20 min were allocated for each scenario. A simple drawing and writing tool was employed, similar to the one applied by Alexander et al. (2012), to initiate discussion, provoke comments and suggestions, allow for a first broad zoning of the study area, and to explore levels of consensus regarding priority areas for change (see Eikelboom and Janssen 2013, Fig. 4a).

**Fig. 4:**
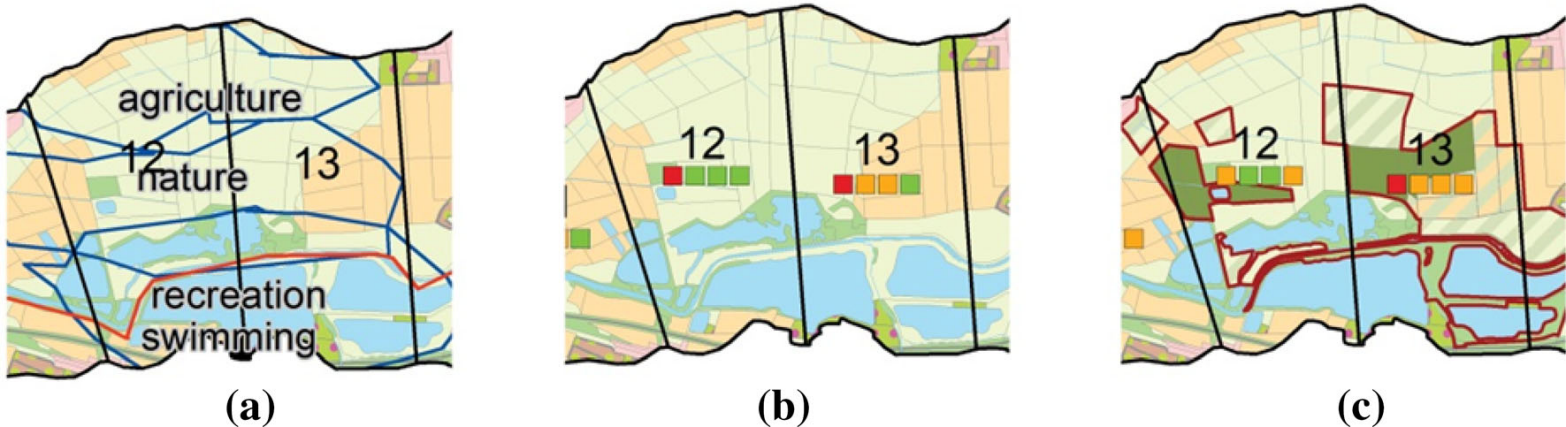
Zoom on segments 12 and 13, **a** scenario sketching: participants outlined priority areas and wrote the annotations using touch table, **b** land use before change with indicator symbols (traffic light), **c** after change of land use (parcels with red borders), e.g. from intensive grassland to forest indicators changed (traffic light), please see Figs. 6 and 7 for the legend

### Step 3: Allocating land uses

In this step, the land use change tool enabled participants to designate land uses assumed to materialise in the study area once the respective scenario was implemented in the future. 50 to 60 minutes were used for each scenario in this step. The tool allowed the selection of one or more predefined land use parcels (Eikelboom and Janssen 2015a) as well as the changing of land use in those parcels. Participants referred to prior ideas generated in the sketching phase and the tasks suggested there for each scenario. Thirteen land use types were selected for application in the Geodesign process, based on input data and scenario requirements. However, the input data contained no information on whether agriculture (cropland or grassland) was intensive or extensive. Therefore, intensive agriculture was set as a default.

### Step 4: Exploring impacts

The fourth step focused on the exploration of impacts of land use changes, and this was accomplished iteratively with the designation of land use changes in the previous step, hence within the 50 to 60 min used for allocating land uses. An impact assessment tool was established in the GIS system whereby impacts were calculated for four selected ecosystem services indicators: food provision, recreation, pollination potential, and climate regulation. We selected the ecosystem services and their indicators based on four premises: (1) they should be sensitive to proposed land use changes as provided by participants on the touch table; (2) they should be complementary to possibly highlight exemplary trade-offs when one indicator is enhanced and another decreased through potential interventions; (3) they should be able to capture the four key types of ecosystem services for people, specifically provisioning, regulating, habitat and cultural services; and (4) they needed to be applicable with limited given data and the highly restricted capacities of the Geodesign system to provide on-the-fly modelling of potential impacts of scenarios on ecosystem services delivery. It was impossible to assess further important impacts, e.g. the contributions of land use changes to alleviating key societal challenges, for two reasons: the number of variables or indicators in the workshop is critical for participants, and the use of three indicators has been shown to be feasible in other Geodesign workshops (Eikelboom and Janssen 2015a, b), and technical feasibility substantially limited the number of indicators to be assessed in real-time. The impact of the land use changes on the respective indicator was visualised as a traffic light (high, medium, low value) for each indicator (Fig. 4b, c), following positive experiences from Eikelboom and Janssen (2015a).

**Fig. 5:**
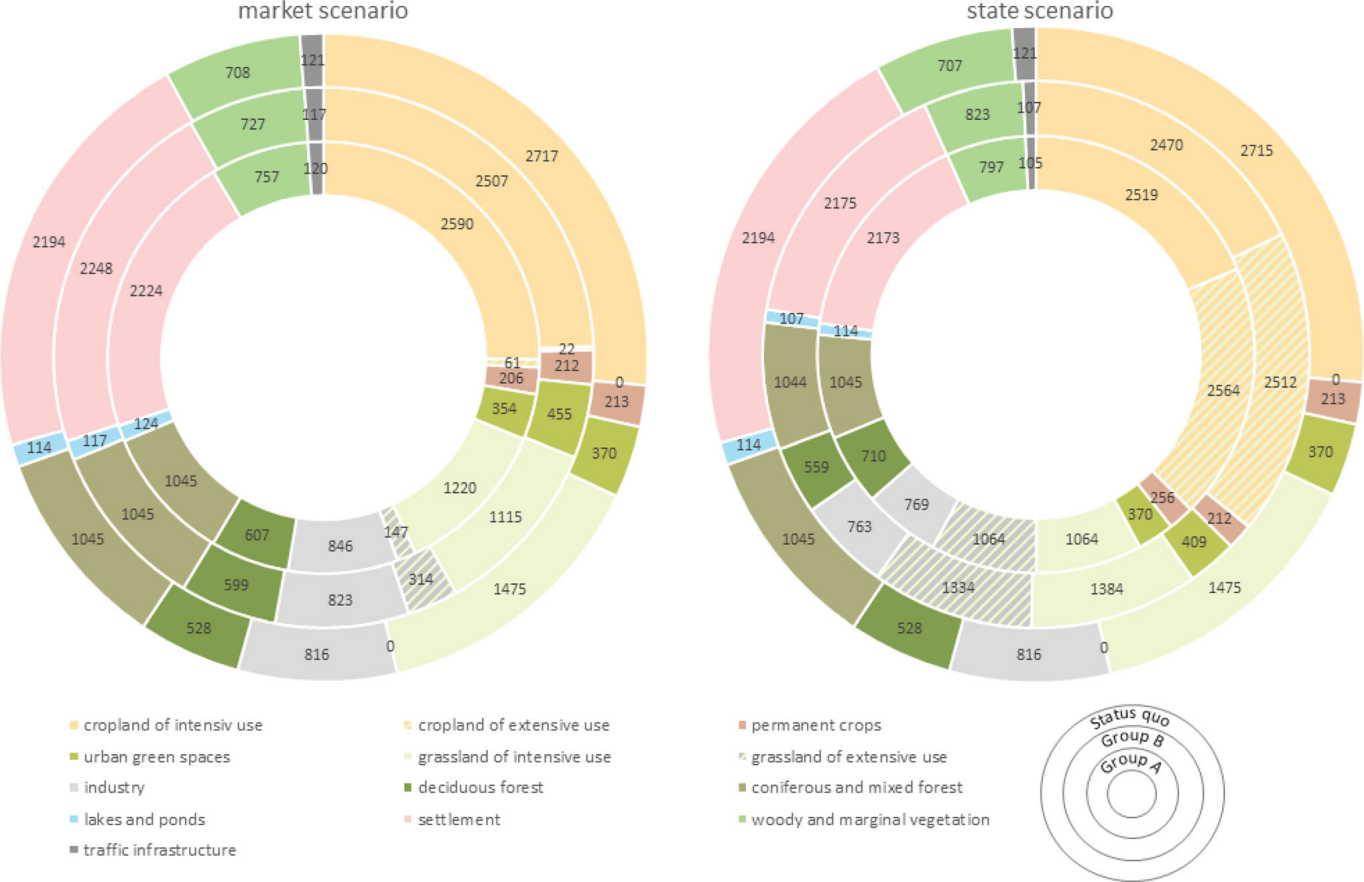
Land use area for each land use in ha; Note: extensive agriculture (cropland and grassland) gained 100% as the initial area was 0 ha

**Fig. 6:**
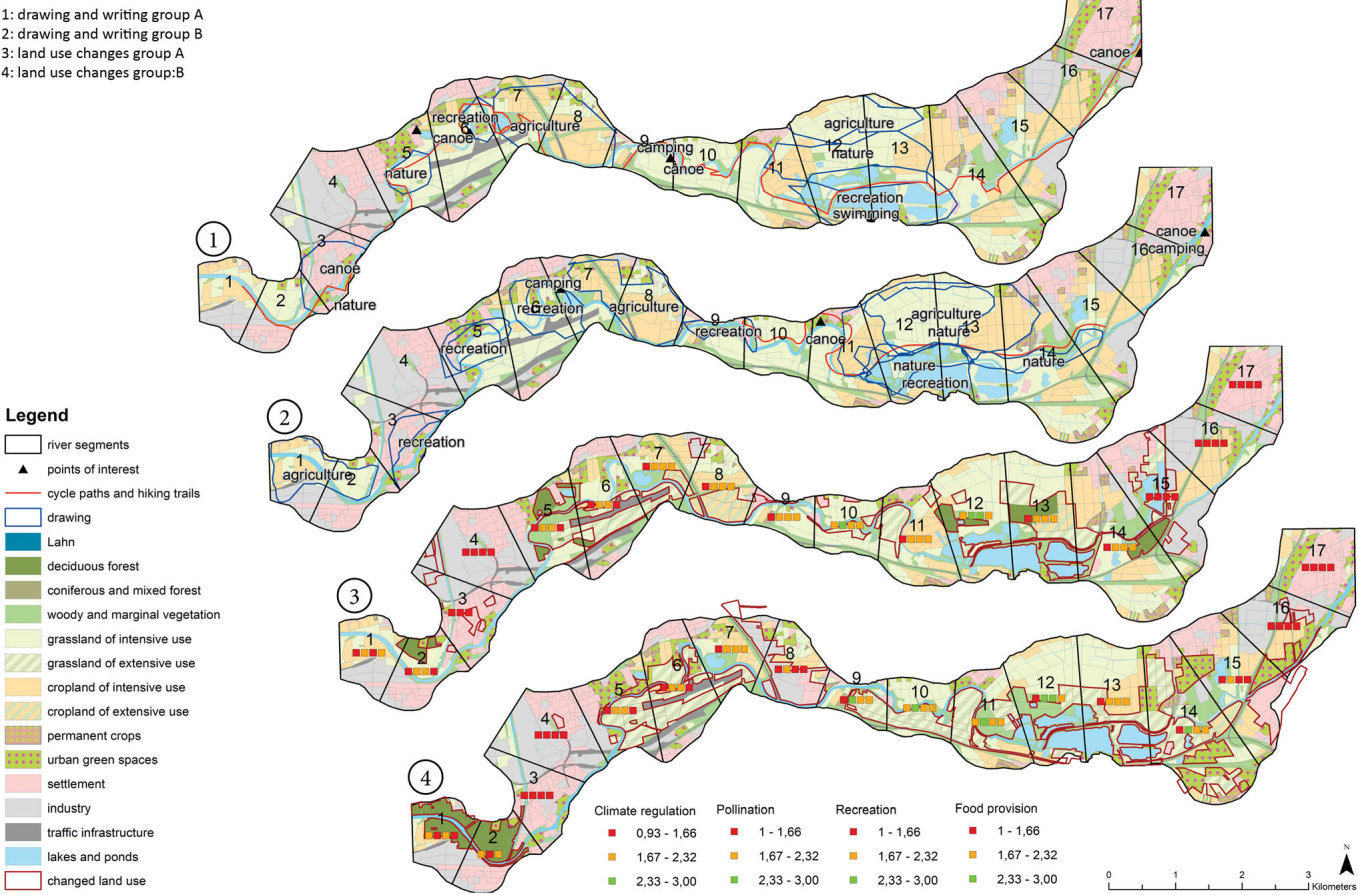
Maps produced in step 2 and 3 showing sketching (maps 1 and 2) and land use change (maps 3 and 4) for Market scenario

**Fig. 7:**
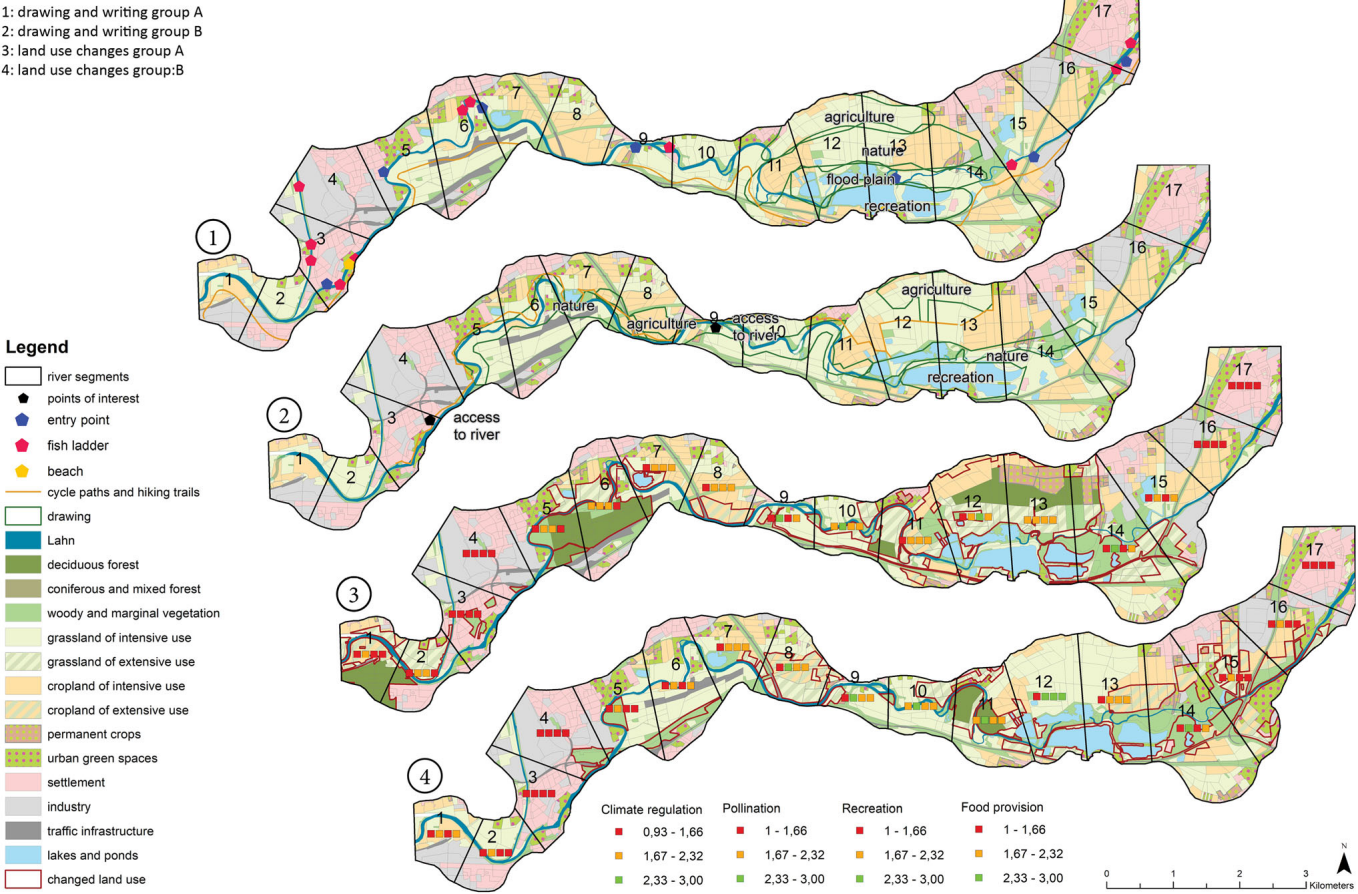
Maps produced in step 2 and 3 showing sketching (maps 1 and 2) and land use change (maps 3 and 4) for State scenario

The impact assessment tool calculated indicator values for each river segment and could be started at any time at participants’ behest. River segments were chosen as the basis for calculation because they appeared to present an acceptable level of aggregation to detect changes, as well as allowing for easy visualisation within the limited calculation time available. Each indicator symbol (traffic light) on the map represented the mean value of the underlying value maps for that river segment (Fig. 5b, c, Supplementary Material S5, S7, S10, S12). Indicators were represented on scales between 1 and 3. Intervals were chosen as thresholds for ‘traffic light’ categories to allow for comparability between indicators and to ease trade-off evaluations. Values between 1 and 1.66 were considered poor (highlighted in red), values between 1.67 and 2.32 represented medium conditions (coloured orange), and values between 2.33 and 3.00 were considered good (represented in green), but participants interacted with colours only. Minimal discrepancies in interval ranges stem from rounding differences. Each land use change undertaken, e.g. from intensive grassland to forest, was considered in the subsequent calculation of the indicator value in the respective river segment. Whilst absolute indicator values changed in relation to the designated land use changes, the traffic lights switched only after the respective thresholds had been surpassed. For this reason, not only the state of traffic lights needs to be taken into consideration, but also the absolute values as calculated. Changes of indicator values of less than .01 are considered very small and are not reported. Some indicator values decreased below 1 after land use changes. As a consequence the traffic light box turned invisible on the map. Therefore, in post-processing, the lowest indicator value had to be adjusted to .93 (Figs. 6 and 7).

### Indicators

The order of the indicators described here follows the order of the indicator boxes shown in the figures, from left to right. Detailed figures and tables can be found in the Supplemental Material.

*Climate regulation* refers to the meaning for climate protection in terms of potential emission or retention of greenhouse gases in the soil. The evaluation is based on a further development of the approach proposed by Saathoff et al. (2013), taking into account soil type and land use and assuming that CO2 emissions cease after five years of ploughing. We chose this method as it allows for a relatively robust estimate of land use change impacts on emissions whilst taking under consideration various combinations of soils and land use types. Based on a classification matrix derived from Saathoff et al. (2013 Supplementary Material, S4) the climate regulation potential for each parcel was estimated; e.g. a parcel used as intensive grassland on humid soil was classified as medium (2), whereas the same land use on a dryer soil type was classified as having low potential for climate regulation (1), (Supplementary Material, S5).

The *pollination* indicator, which represents suitable habitats for pollinating species (especially insects such as bees and butterflies), corresponds largely to the general meaning of the habitat for flora and fauna. It has been evaluated based on land uses considering three factors: light, vegetation diversity, and the ratio of sealed surface (Hausmann et al. 2011, 2016). The evaluation of each land use based on these factors was conducted by three scientists with backgrounds in environmental science, geography and environmental planning. In terms of the climate regulation indicator, classes ranged from high (3) to low (1), (Supplementary Material S6, S7).

The third indicator represents the (used) availability of *nature-based recreation*. The evaluation takes into account landscape aesthetics, specifically perceived naturalness based on land use data (Walz and Stein 2014; Hermes et al. 2018), and actual recreational use. The data for the last category were generated in a public participation GIS (PPGIS) survey in 2017. The density of recreational places was visualised (see e.g. Laatikainen et al. 2015) and combined with perceived naturalness, based on a matrix. Values range between high (3) and low (1); for example, high perceived naturalness and medium recreation density yield a high value for nature-based recreation (Supplementary Material S8–S10).

Finally, *food provision* represented the importance of that area for the agricultural production of food, fodder or raw material. The evaluation was based on the intensity of the agricultural use and land use data. Intensive agriculture, permanent crops and intensive grassland were expected to yield a high value for food provision, whilst extensive agriculture and extensive grassland yielded a medium value for food provision (3). All other land uses yielded a low value for food provision (1), (Supplementary Material S11, S12).

### Step 5: Reflecting

In the final step stakeholders discussed (for 30 minutes) and evaluated (for 15 minutes) the workshop in a plenary session, in the course of which they identified positive and negative aspects. Participants further evaluated the workshop using a “suitcase-question mark-garbage bin” method, where they wrote down what they were taking away (positively) from the workshop (“the suitcase”), the remaining questions (“the question mark”), and unnecessary or negatively perceived elements of the workshop (“the garbage bin”). Following this they answered questions in a mini-survey reflecting their workshop experience. The entire session was chaired by a professional facilitator.

### Methods applied to assess Geodesign contributions to planning with NBS and boundary management

The co-produced spatial NBS scenarios or boundary objects are described in terms of the differences between scenarios (State vs. Market), stakeholder groups (A vs. B), tools (sketching vs. land use change), and touch interfaces (large vs. small). To do so, we compared the amount, size, distribution, and type of drawings/writings and land use changes. The comparison was possible due to (1) the equal distribution of participants amongst the two groups, also in terms of institutional affiliation and prior workshop participation, and (2) the use of both interfaces by both groups (Group A worked on the Market scenario with the large interface and, on the State scenario, with the small interface, and vice versa).

In regard to the facilitation of the boundary management functions and the impact on perceived credibility, salience and legitimacy, we used a mixed-methods approach consisting of qualitative/narrative data (observations, feedback discussion and evaluation exercise) and numerical data (mini-surveys) provided by participants (Tashakkori et al. 2015) (Table 1). Observations were noted during the workshop by one researcher per stakeholder group. Participant observation enables the qualitative assessment of “explicit culture”, that is, “what people are able to articulate about themselves”, and “tacit” aspects, that is, aspects that “remain outside our awareness or consciousness” (Musante and DeWalt 2010, p. 1). In this case, researchers observed how the Geodesign workshop participants interacted with the tools and with each other to develop the spatial NBS scenarios. Additionally, all researchers were asked to reflect upon their observations immediately after the workshop. Furthermore, a survey asked participants to rate their degree of agreement with statements on perceived gains in practical, target, and transformation knowledge (Adler et al. 2018) using a 5-point Likert scale. Finally, participants made use of moderation cards to communicate specific feedback on positive and negative aspects, as well as their questions regarding the workshop content and design (Supplementary Material S12).

**Table 1:** Boundary management evaluation guide (based on Cash et al. 2003; Clark et al. 2016)

Functions	Explanation	Guiding questions
Translation	1. Translation from individual thoughts/knowledge values into spatially explicit information 2. Translation of the story line into tasks guiding spatial scenario development	1. How did the participants handle the Geodesign tools?; How did the participants reflect/comment on the tools? 2. To which extend did the participants implement the tasks?; How did the participants reflect/comment on the tasks?
Communication	1. Communication between and/or amongst participants	1. How did the Geodesign process facilitate communication? 2. How did the participants evaluate the process for communication/group work?
Mediation	1. Mediation of participants’ views through the tool (feedback) 2. Mediation of participant opinions through a professional mediator	1. Did the tool feedback system (indicator) mediate between the participants’ views? (observation) 2. Was there a need for mediation? How did it show?
Criteria		
Credibility	Perceived level of scientific credibility of the Geodesign process and results	To what degree do participants seem to perceive the Geodesign process and output to be technically adequate in handling of evidence?
Salience	Perceived level of practical relevance of the Geodesign process and results	To what degree do participants seem to perceive the Geodesign process and output to be relevant to the decision or policy?
Legitimacy	Perceived level of political legitimacy of the Geodesign process and results	To what degree do participants seem to perceive the Geodesign process and output to be fair, unbiased, respectful of all stakeholders?

## Results

### Spatial scenarios of the future Lahn river landscape

For each scenario, Geodesign workshop participants created one sketch map and one land use allocation map. The results illustrate substantial differences between maps created by different groups, with different tools, and with touch tables of different sizes. A more detailed description of the produced maps in each scenario can be found in Supplementary Material Box 2.

In the Market scenario, more priority areas were drawn and hence more areas (813 ha in Group A, 1200 ha in Group B) were prioritised for purposes other than those in the State scenario (506 ha in Group A, 460 ha in Group B). In the State scenario, both groups allocated most priority areas (184 ha in Group A, 256 ha in Group B) to nature conservation (Figs. 6 and 7). In the State scenario, more land use was changed (455 ha in Group A, 714 ha in Group B) than in the Market scenario (360 ha in Group A, 639 ha in Group B). In both scenarios, predominantly intensive agriculture was converted into other land uses, and extensive grassland was the chief type of new land use. Traffic infrastructure and industry decreased to a greater degree in the Market scenario (Fig. 5).

However, differences exist between Group A and Group B in terms of the spatial translation of the scenarios. In the Market scenario, Group B allocated most priority areas to recreation (five out of ten), whereas Group A chose nature conservation (three out of seven, Fig. 6). Similarly, in both scenarios Group B increased the amount of urban green (by 85 ha, and 39 ha in the Market and State scenarios, respectively), whereas Group A decreased the area for urban green spaces in the Market scenario from 370 ha to 345 ha. Group A was more prone to allocating deciduous forests (79 ha and 182 ha in the Market and State scenarios, respectively) than Group B (71 ha and 31 ha in the Market and State scenarios, respectively, Figs. 5 and 7).

In regard to comparing tools, in the Market scenario participants used the drawing tool to sketch almost double the area (813 ha in Group A, 1200 ha in Group B) than they actually changed using the land use change tool (360 ha in Group A, 639 ha in Group B); this, however, was not the case in the State scenario. At the small touch table *the size* of priority areas was larger in both scenarios than at the large table. Yet *the number* of priority areas was smaller than at the large table in the State scenario. In both scenarios, more land use was converted at the smaller table, which also had an effect on the indicators (see Supplementary Material S13).

### Evaluating boundary management functions in Geodesign

#### Indications of Geodesign contributions to ‘Translation’

The Geodesign tools appeared well-suited to facilitate translation of individual ideas and knowledge into spatial representations. Participants readily became acquainted with tools following a short introduction. Despite lack of prior experience, participants easily overcame the technical obstacles of the touch screen (e.g. drawing with a touch pen or finger) and showed high motivation for experimentation. The resulting maps represent jointly produced spatial translations of scenario stories that can clearly be interpreted as boundary objects.

Participants used the tasks as a guide to develop the spatial scenarios, and they fulfilled these partially. The tasks were discussed critically: prior to beginning the drawing process, participants in both groups debated the scenario outline and the resulting tasks, in particular the question of how realistic the tasks were. The amount of extensive agriculture respondents were to allocate was found to be too high—a fact reflected in the groups’ completion of this task. In both scenarios, the groups missed the stated target for converting agricultural land from intensive use to extensive use. In other tasks, participants exceeded task goals; in the Market scenario, for example, participants in both groups allocated almost twice the required area of forest. The number and complexity of the tasks was clearly criticised by the participants (“Time short (tasks too large)”, “Tasks for group work should be less extensive”, Supplement Material S14).

The usefulness of the predefined specific tasks was extensively debated. On the one hand, participants acknowledged that tasks such as percentage targets aided the participatory planning process and facilitated the translation of scenario stories into spatial maps within a short period of time. On the other hand, participants argued that the predefined targets and limited time available did not realistically reflect real-world planning situations, where more discussion would be possible. Furthermore, the tasks were seen as limiting participants’ creativity. One participant argued that the scenarios would have been implemented more satisfactorily without the tasks, using experiences generated in planning processes (Tables 2 and 3).

**Table 2:**
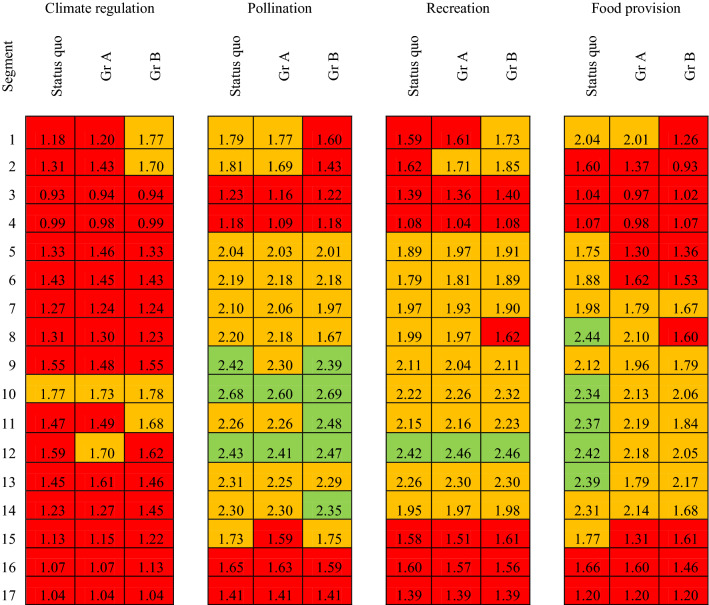
Summary table of the four indicators for each river segment for the status quo and Market scenario land use changes of group A and B. Traffic lights representing indicator values for each indicator, green: high value (2.33–3.0), orange: medium value (1.67–2.32), red: low value (1–1.66); values below stem from a minor computational error

**Table 3:**
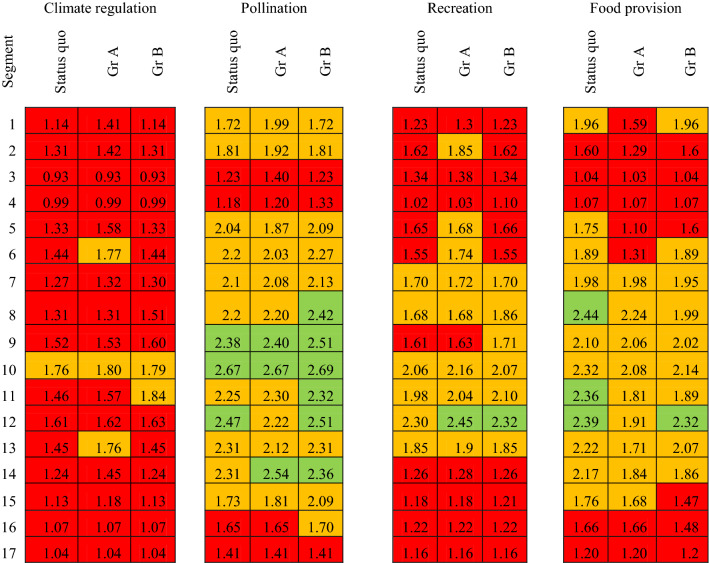
Summary table of the four indicators for each river segment for the status quo and State scenario land use changes of group A and B. Traffic lights representing indicator values for each indicator, green: high value (2.33–3.0), orange: medium value (1.67–2.32), red: low value (1–1.66)

#### Indications of Geodesign contributions to ‘Communication’

Communication was facilitated by each tool in the Geodesign process. In particular, the task of translating scenario stories into spatial maps highlighted the need for further debate and clarification. For example, one group debated potential sizes and locations of priority areas for nature, agriculture and recreation. Another group discussed whether changes should be small and parcelled, following the scenario narrative, or instead include larger connected areas. After one influential participant had proposed avoiding agricultural land use close by the river due to risks of nutrient input and erosion, the group developed the idea of developing a network of nature protection areas along the floodplain and locating agriculture only in areas farther from the river. Although some participants took the lead in the actual drawing, these individuals did not dominate the discussions and all participants jointly decided on the locations. Numerous positive comments were made regarding internal group communication during the workshop; one participant designated Geodesign as a good “communication tool”. Accordingly, the survey results show that, on average, participants believed that the workshop supported the exchange and collaboration between LiLa partners (*M* = 4.2, Table 4). Nevertheless, one participant pointed out that the workshop provided “[…] too little time for exchange with participants”. Whilst the workshop was designed to support communication, its tight scheduling inhibited exchange.

**Table 4:** Mean value of statements about practical, target and transformation knowledge rated on a 5-point Likert scale by workshop participants, *N* = 11, Likert scale: 1 = I fully disagree, 2 = I rather disagree, 3 = I don’t have a solid opinion, 4 = I rather agree, 5 = I fully agree

Statement	M
I gained knowledge on how the interplay between important factors impact the future development of the Lahn river landscape	3.4
I gained knowledge on possible options for action for future development of …	3.3
I gained knowledge on how different actions could be realised in practise	2.9
I feel better prepared to take part in discussions and decision processes on a sustainable development of the Lahn river landscape	3.5
The workshop supported the exchange and collaboration between Lila partners	4.2
The employed technology is potentially useful for supporting participatory planning processes in river landscapes	4.5
I am interested to use a further developed version of this technology in our upcoming work of the Lila project	4.4

#### Indications of Geodesign contributions to ‘Mediation’

The ‘feedback system’ tool of the Geodesign process could have been particularly helpful in facilitating the mediation of various ideas by providing insights into likely impacts of scenarios. However, the tool’s practical functionality in facilitating mediation was limited by technical challenges, such as the small number of indicators considered (“which do not sufficiently reflect the complexity of ecosystem services”), the calculation time required (about thirty seconds), and low sensitivity to proposed land use changes. Participants became frustrated (“That is frustrating!”, “I don’t believe it [the indicator values], the computer lies!”), and started to question the credibility of the Geodesign process (“Who sets up the process, has it in their hands.”). This notwithstanding, the general concept of applying real-time scenario impact assessments using indicators was embraced by participants, and the statement regarding knowledge gains in regard to the interplay between important drivers of future development was evaluated slightly positively (*M* = 3.4, Table 4).

The facilitators involved in the Geodesign processes adopted further mediating roles. In other words, facilitators encouraged participants to start playing with the tool, or they helped when participants encountered misunderstandings regarding tasks or difficulties in operating the GIS system. For example, participants would coincidently touch the screen and thereby activate functions (such as moving the map, zooming, etc.) they were unable to revoke. Beyond this, facilitators strove to keep everyone aware of important side discussions, and they addressed minor power issues and ensured that all participants had equal opportunities to engage with the tool. For example, one influential participant questioned another participant’s choice in allocating priority areas, which had an intimidating effect that resulted in lower participation by that participant.

#### Stakeholder perceptions of Geodesign credibility, salience and legitimacy

The perceived credibility of the Geodesign process suffered chiefly from discontent with indicator selection and functioning, as well as time management. Further, participants questioned data sources, such as the location of swimming spots, the geographic extent (“Too many river segments, therefore confusing and too much”), and the limited land uses from which to choose. They highlighted that the available data were insufficient for them to fully engage with local complexity. For example, data on the current floodplain, satellite images, and additional and more detailed land uses (e.g. integrated riparian forest) would have been beneficial.

In general, participants perceived the Geodesign process as salient to local concerns and issues, despite critique voiced in regard to the indicators. For example, they interpreted the Geodesign process as an “appropriate communication tool; talk, ‘draw’, evaluate quickly in one process”. Participants perceived the Geodesign process as generally useful for their own work (high to very high agreement; see Table 4), especially in terms of engaging citizens in plans for new development locations.

The overall atmosphere was perceived by both the researcher and participants as being very positive and peaceful (“[…], good workshop atmosphere”). The participants knew each other and the research team from previous workshops.

## Discussion and conclusion

The study showed that participatory Geodesign processes can readily be applied to develop spatial scenarios for planning with NBS. Furthermore, the Geodesign process proved generally useful in facilitating boundary management: (1) scenario stories were successfully, if variously, *translated* into spatial NBS scenarios by the two stakeholder groups; (2) the process facilitated fruitful discussion and was perceived as useful for *communication*; however, (3) *mediation* using a more complex indicator tool led to frustration and a decrease in trust.

### Spatial scenarios of the future Lahn river landscape

#### Geodesign for NBS planning

The Geodesign process presented here was employed as a specific form of participatory scenario planning which offered specific tools that enabled workshop participants to include information on impacts and trade-offs in their decisions. It has shown itself to be a strong method for enabling dialogue between local and technical knowledge and strengthening relationships between local stakeholder and scientists. The process was designed to provoke a co-learning process (cross-fertilisation) and creative thinking (Oteros-Rozas et al. 2015). Additionally, the impact assessment of exemplary indicators in this study has shown how Geodesign supports the identification of co-costs and co-benefits, which is a core principle of NBS planning (Raymond et al. 2017; Cohen-Shacham et al. 2019). For example, the change from intensive grassland to extensive grassland and forest led to an increase of climate change regulation potential and recreation and, concomitantly, a decrease in pollination and food provision values (‘strong State’ scenario, Group A, segment six). This shows that the transformation of scenario narratives into spatial scenarios opens up new possibilities for assessing and evaluating NBS and can be seen as a means for engaging diverse stakeholders in co-producing maps of their shared space, or boundary.

Whilst the term ‘NBS’ was not explicitly employed during the workshop with the participants, the workshop organiser had prepared the process with the intention of steering towards the design of NBS measures. The design of each measure’s specific details was beyond the scope of the workshop. Some measures lead to NBS for floodplain restoration and management (NWRM 2015; Guerrero et al. 2018; Davies and Lafortezza 2019), such as the allocation of forest in the floodplain (NWRM 2015). Participants changed the agricultural areas predominantly from intensive grassland or cropland to extensive use thereof. This extensive use could be operationalised by low tillage agriculture, green cover, early sowing, reduced stocking density, or targeted planting for ‘catching’ precipitation (NWRM 2015). Furthermore, participants allocated fish ladders to create opportunities for fish migration (Guerrero et al. 2018). Points of accessibility to the river allocated by the participants presents a further NBS (Guerrero et al. 2018), as the European Commission (2015) has highlighted also for green areas.

The results differed between the scenarios, both of which had contrasting underlying governance and business models for the planning and implementation of NBS (the thrid criteria in Albert et al. 2019). Participants assumed that a strong government would support more numerous and larger nature priority areas than would market-driven development, even though both have high priorities for collaboration with nature. This can be explained by the fact that, in Germany, nature conservation is a legal obligation for governments from the national to community levels, as stipulated by the Federal Nature Conservation Act (Federal Ministry of Justice and Consumer Protection 2009). However, new alternatives to traditional landscape planning exist today, such as landscape stewardship driven by the action of people (Opdam 2017), or Green Bonds (Flammer 2019). It is striking that the designs differed between the groups, with one group showing more dedication to recreation whilst the other promoted nature conservation. The groups were assembled in such a way as to equally represent the LiLa member institutions as well as previously highlighted boundaries. Nevertheless, all participants are individuals whose behaviour naturally cannot be predicted. It has been shown that deliberative processes can reduce consistency between an individual’s attitudes and their behaviour (Ryfe 2005). The differences between the tools (drawing and land use change) can be explained by the inherent characteristics and ease of use. Participants could draw freely and needed only to touch the map and move the finger indicating the polygon. In contrast, to perform the land use change operation participants had to select a type of land use and a number of parcels on the map. It stands to reason that we observed more hesitation during this task.

#### Geodesign facilitating the co-production of boundary objects

These co-produced scenario maps can be referred to as boundary objects because they are commonly produced and can be used further by the stakeholders as they provide the characteristic interpretive flexibility and were created out of common necessity (Star 2010). In this study, we used a digital Geodesign process. Yet, any form of map in collaborative workshops provides a shared platform for exchanging ideas (Carton and Thissen 2009). Before the mass availability of digital tools (and even today in some contexts) workshops were supported using large hard A0 copies of generally topographical maps. Marker pens were used to draw designs on sheets of tracing paper or transparencies (Albert et al. 2015; Burrough et al. 2015). The paper map approach has clear advantages, for it is easy to use and flexible. Digital Geodesign processes are based on the same principles yet enable the use of multiple map layers and zoom functions, as well as providing and producing geo-referenced information. In addition, digital Geodesign tools can provide immediate feedback to participants by calculating the expected impact of user interventions.

There is a reason to argue that boundary objects extend further than developed maps. Maia (2010, p. 1240) states that the participatory process itself could be seen as a boundary object, where different stakeholders “temporarily align themselves around a common project for the purpose of development implementation”. Whilst making changes to the maps, participants reflect on these maps, answer questions and discuss the changes they make. The map can be seen as a manifestation of these reflections and discussions. In this way, workshop notes become an integral part of the results. Observations and survey results provide background to the process of map generation and hence become crucial elements for understanding the results.

### Evaluating boundary management functions in Geodesign

#### Geodesign fulfilling the functions of boundary management

Participants used the tools to *translate* their ideas and the provided tasks related to previously established scenarios into spatial maps. In contrast to other scenario-based planning studies (e.g. Wissen Hayek et al. 2016), the translation was performed by the participants themselves. This has also been shown to work in other contexts, such as climate change adaptation strategy planning (Eikelboom and Janssen 2015a), marine spatial planning (Janssen et al. 2014), clean neighbourhood energy planning (Hettinga et al. 2018), wind turbine planning (Rafiee et al. 2018), and in educational contexts (Albert et al. 2015). The fact that both groups translated the scenario stories differently into spatial maps demonstrates the importance of using maps, and specifically Geodesign, to make ideas and visions concrete. Mapping allows and simultaneously forces users to be precise in terms of location, size and form – within a geographic context. Differently from other design models which rely on internal coordinates, Geodesign models or process outputs are integrated into greater spatial contexts as a GIS project (Steiner and Shearer 2016).

The boundary management function of *communication* occurred in a variety of ways: (1) local information was communicated from the stakeholders to the researcher; (2) scientific information was communicated from the researcher to the local stakeholder; and (3) different views, interests and values were communicated between the stakeholders themselves. Cross-fertilisation amongst these diverse knowledge systems “can contribute new evidence and also improve the capacity to interpret conditions, change, responses, and in some cases causal relationships in the dynamics of social-ecological systems” (Tengö et al. 2014, p. 580). In our Geodesign process, such new evidence was co-produced in form of boundary objects.

Both the impact assessment tool as well as the Geodesign facilitators were assumed to *mediate* the design process. However, the impact assessment tool was perceived by participants as the weakest component of the workshop. Technical inabilities of the tool and the complexity of the indicators led to frustration and diminished trust in the tool. In line with this, previous studies comparing different Geodesign tools that enable immediate feedback found that tools with high information levels or high levels of aggregation were rated by participants as being the least useful for communication (Arciniegas et al. 2013; Eikelboom and Janssen 2015b). The workshop presented here reveals a need to test indicators and their thresholds for change. One participant critically referred to the power of the researchers in setting up the tool and deciding on these values, thus raising the issue of legitimacy. Comparing the change the traffic light symbol with changes of absolute indicator value revealed that certain changes remained invisible for participants whilst other, smaller changes led to a change of colours in the traffic light system. Future applications in the NBS context should also seek ways in which to assess indicators that better address the impacts of interventions in regard to identified societal challenges.

The perceived credibility, salience and legitimacy were overall acceptable, but suffered from the complexity of the indicator assessment tool and data availability. Credibility in the process has been shown to depend largely on its technical components (Adem Esmail and Geneletti 2017) or an assessment methods as in the presented workshop. These technical components were the most controversial because they were seen as very positive for communication, yet as negative when complexity increased. The scenarios used in the Geodesign process at hand were developed by the participants in a prior workshop, thereby positively affecting the salience of the process as they were tailored to the needs of the participants (van Oudenhoven et al. 2018). Whilst it is not fully possible to conclude that the generated information was unbiased and respected the diversity of values (Clark et al. 2016), the positive workshop atmosphere allowed for such a process. An additional factor here was the presence of a professional facilitator who mediated the process to cope with power relations and different stakeholder values (Reed et al. 2017).

#### Boundary management conceptually framing Geodesign

This paper also contributes to the development of a general concept of Geodesign by further exploring the idea of Geodesign as a tool with which to facilitate boundary management. Current research is more substantive in nature—presenting tools and highlighting insights from distinct case studies—rather than comprehensive or contributing to the general concept of Geodesign (Campagna 2015). A conceptual framing of Geodesign that employs a well-established social science concept could potentially increase its applications in practice by providing a clear and structured understanding which can be detached from specific cases. This answers a call by researchers who have highlighted the mismatch between the provision of tools and their actual use in practice (Vonk et al. 2007; Vonk and Geertman 2008; Currier and Couclelis 2014; Pelzer et al. 2015a, b).

### Recommendations for future Geodesign studies for NBS planning

The workshop process presented here was embedded in both a transdisciplinary project and a specific socio-environmental and political context. Yet, it can be applied to other case studies because its design was also inspired by previously conducted Geodesign workshops (Alexander et al. 2012; Janssen et al. 2014), which were characterised by different contexts. In a workshop in Friesland in the Netherlands, local residents designed spatial solutions to reduce soil decline in fen meadow areas (Janssen et al. 2014). Geodesign was also applied to allocate tidal devices around the Mull of Kintyre in Scotland (Alexander et al. 2012). Participants in those workshops were local stakeholders, such as fishermen, yacht owners, tourist operators, and tidal energy developers. Geodesign has also been used to develop a regional plan for the Lower Zambezi Region in Mozambique (Janssen and Dias 2017). Participants were local stakeholders representing the various economic sectors of the region, such as mining, tourism, agriculture and fisheries. As opposed to the Lahn workshop, none of these workshops’ participants had any previous interaction.

In conclusion of this workshop, we wish to present the following design recommendations:

- Tools should be as simple as possible. Tools should limit the amount of information to be processed by participants. Special attention must be paid to map design as multiple types of information need to be presented on a single map. The use of uniform legends and traffic lights to present indicator values superimposed on land use maps proved to be effective. The higher the complexity (e.g. integrating impact evaluation tools), the greater the need to co-design (parts of) the Geodesign process with the stakeholders.
- Organising Geodesign workshops is a learning process that requires substantial effort in terms of preparation, logistics and technical challenges. Preparation is especially crucial for there should be no technical problems, as experienced in the presented workshop, and all relevant information must be readily available. Participants evince little tolerance for technical or methodological errors.
- Selection of the participants is essential. To avoid self-selection bias, participation should be by invitation only. It is important that the workshop has benefits for the participants, such as contact to other stakeholders and access to new information, and that the workshop is a positive experience, as was appreciated also by participants in this workshop.
- The success of the workshop depended highly on the cooperative attitude of the participants. This requires consensus-oriented ways of decision-making. However, it is uncertain if the same approach would also work in contexts of sharp conflict or with a more power-based style of decision-making.
- Working with several groups can be beneficial as it provides additional opportunities for considering a broader set of opinions. In our workshop, we thereby collected surprising insights that might not have surfaced in just one joint session.

All in all, we conclude that the translation of scenario narratives into spatial maps deepens the understanding and details of the developed NBS scenarios, thereby enabling the identification and evaluation of co-costs and co-benefits. Furthermore, Geodesign has proved useful in supporting the functions of boundary management (translation, communication, mediation), but the increasing complexity seemed to compromise the perceived credibility and legitimacy of the process. Further research and practical experimentation could revolve around the co-development of impact assessment tools with stakeholders (e.g. choice of indicators), adjustments to indicator visualisation, and the adaptation of time management according to the number of workshop tasks. Geodesign can be used in all phases of a decision process, although it is usually employed in the identification and development phase. Developing planning alternatives is a crucial and complex step in each decision process, and the results determine to a large extent which final decision will be implemented. The Geodesign process presented in this contribution generated maps which may define the boundaries for the final decision-making on sustainable river landscape concepts. Policy makers should harness opportunities for applying transdisciplinary spatial planning processes so as to integrate diverse perspectives and co-generate knowledge relevant for realising more sustainable river landscape development that provides benefits to people and the natural environment.

## Electronic supplementary material

Below is the link to the electronic supplementary material.

Electronic supplementary material 1 (PDF 717 kb)

## References

[CR8] Adem Esmail B, Geneletti D (2017). Design and impact assessment of watershed investments: An approach based on ecosystem services and boundary work. Environmental Impact Assessment Review.

[CR1] Adler C, Hirsch Hadorn G, Breu T, Wiesmann U, Pohl C (2018). Conceptualizing the transfer of knowledge across cases in transdisciplinary research. Sustainability Science.

[CR2] Albert C, von Haaren C, Vargas-Moreno J, Steinitz C (2015). Teaching scenario-based planning for sustainable landscape development: An evaluation of learning effects in the Cagliari Studio Workshop. Sustainability.

[CR3] Albert C, Schröter B, Haase D, Brillinger M, Henze J, Herrmann S, Gottwald S, Guerrero P (2019). Addressing societal challenges through nature-based solutions: How can landscape planning and governance research contribute?. Landscape and Urban Planning.

[CR4] Alexander KA, Janssen R, Arciniegas G, O’Higgins TG, Eikelboom T, Wilding TA (2012). Interactive marine spatial planning: Siting tidal energy arrays around the mull of kintyre. PLoS ONE.

[CR5] Arciniegas G, Janssen R, Rietveld P (2013). Effectiveness of collaborative map-based decision support tools: Results of an experiment. Environmental Modelling & Software.

[CR6] Barth N-C, Döll P (2016). Assessing the ecosystem service flood protection 
of a riparian forest by 
applying a cascade approach. Ecosystem Services.

[CR7] BKG. 2016. Digital Basic Landscape Model (AAA Modelling) Basic-DLM (AAA), 1–62.

[CR9] BMUB and BfN (2009). Auenzustandsbericht: Flussauen in Deutschland.

[CR10] Burrough PA, McDonnell RA, Lloyd CD (2015). Principles of geographical information systems.

[CR11] Campagna, M. 2015. Geodesign as a process: From modelling to enactment. *In Proceedings of Digital Landscape Architecture*, pp 276–283.

[CR12] Carton LJ, Thissen WAH (2009). Emerging conflict in collaborative mapping: Towards a deeper understanding?. Journal of Environmental Management.

[CR13] Cash DW, Clark WC, Alcock F, Dickson NM, Eckley N, Guston DH, Jager J, Mitchell RB (2003). Knowledge systems for sustainable development. Proceedings of the National Academy of Sciences.

[CR14] Clark WC, Tomich TP, van Noordwijk M, Guston D, Catacutan D, Dickson NM, McNie E (2016). Boundary work for sustainable development: Natural resource management at the Consultative Group on International Agricultural Research (CGIAR). Proceedings of the National Academy of Sciences.

[CR15] Cohen-Shacham E, Andrade A, Dalton J, Dudley N, Jones M, Kumar C, Maginnis S, Maynard S (2019). Core principles for successfully implementing and upscaling Nature-based Solutions. Environmental Science and Policy.

[CR16] Currier K, Couclelis H, Lee DJ, Dias E, Scholten HJ (2014). Geodesigning ‘From the Inside Out’. Geodesign by integrating design and geospatial sciences.

[CR17] Davies C, Lafortezza R (2019). Transitional path to the adoption of nature-based solutions. Land Use Policy.

[CR18] Editorial N (2017). Natural language: The latest attempt to brand green practices is better than it sounds. Nature.

[CR19] Eikelboom T, Janssen R (2013). Interactive spatial tools for the design of regional adaptation strategies. Journal of Environmental 
Management.

[CR20] Eikelboom T, Janssen R (2015). Collaborative use of geodesign tools to support decision-making on adaptation to climate change. Mitigation and Adaptation Strategies for Global Change.

[CR21] Eikelboom T, Janssen R (2015). Comparison of Geodesign tools to communicate stakeholder values. Group Decision and Negotiation.

[CR22] Ervin, S. 2011. A system for GeoDesign. *In Proceedings of Digital Landscape Architecture*, pp 145–154.

[CR23] European Commission. 2015. *Towards an EU Research and Innovation policy agenda for Nature-Based Solutions & Re-Naturing Cities*. Final report of the Horizon 2020 expert group on ‘Nature-based solutions and re-naturing cities’. Brussels.

[CR24] Federal Ministry of Justice and Consumer Protection. 2009. *Federal Nature Protection Law*. Germany.

[CR25] Flammer C (2019). Corporate Green Bonds. Academy of Management Proceedings.

[CR26] Fliervoet JM, Van den Born RJG, Smits AJM, Knippenberg L (2013). Combining safety and nature: A multi-stakeholder perspective on integrated floodplain management. Journal of Environmental Management.

[CR27] Gottwald S, Janssen R, Raymond C, Sang N (2020). Can Geodesign be used to facilitate boundary management for planning and implementation of nature-based solutions?. Modelling nature-based solutions.

[CR28] Guerrero P, Haase D, Albert C (2018). Locating spatial opportunities for nature-based solutions: A river landscape application. Water.

[CR29] Hausmann A, Haszprunar G, Segerer AH, Speidel W, Behounek G, Hebert PDN (2011). Now DNA-barcoded: The butterflies and larger moths of Germany (Lepidoptera: Rhopalocera, Macroheterocera). Spixiana.

[CR30] Hausmann A, Slotow R, Burns JK, Di Minin E (2016). The ecosystem service of sense of place: Benefits for human well-being and biodiversity conservation. Environmental Conservation.

[CR31] Henze J, Schröter B, Albert C (2018). Knowing me, knowing you-capturing different knowledge systems for river landscape planning and governance. Water (Switzerland).

[CR32] Hermes J, Albert C, von Haaren C (2018). Assessing the aesthetic quality of landscapes in Germany. Ecosystem Services.

[CR33] Hettinga S, Nijkamp P, Scholten H (2018). A multi-stakeholder decision support system for local neighbourhood energy planning. Energy 
Policy.

[CR34] HVBG, 2016. Cartographic Service of the Hessian Administration for Soil Management and Geoinformation.

[CR35] Janssen R, Arciniegas G, Alexander KA (2014). Decision support tools for collaborative marine spatial planning: Identifying potential sites for tidal energy devices around the Mull of Kintyre, Scotland. Journal of Environmental Planning and Management.

[CR36] Janssen R, Dias E (2017). A pictorial approach to Geodesign: A case study for the Lower Zambezi valley. Landscape and Urban Planning.

[CR37] Janssen, R., Eikelboom, T., Verhoeven, J., and Brouns, K. 2015. Using Geodesign to develop a spatial adaptation strategy for Friesland. *In*: Proceedings of the *Geodesign by integrating design and geospatial sciences*, pp 103–116.

[CR38] Laatikainen T, Tenkanen H, Kyttä M, Toivonen T (2015). Comparing conventional and PPGIS approaches in measuring equality of access to urban aquatic environments. Landscape and Urban Planning.

[CR39] Lafortezza R, Chen J, van den Bosch CK, Randrup TB (2018). Nature-based solutions for resilient landscapes and cities. Environmental Research.

[CR40] LiLa. 2019. EU-LIFE-Project LiLa—Living Lahn—One river, many interests—Concepts and measures for a region worth living [online]. Retrieved November 12, 2019, from https://www.lila-livinglahn.de.

[CR41] Maia G (2010). Making development agents: Participation as boundary object in international development. Journal of Development Studies.

[CR42] Musante, K., and DeWalt, B. 2010. *Participant observation: A guide for fieldworkers*.

[CR43] Nesshöver C, Assmuth T, Irvine KN, Rusch GM, Waylen KA, Delbaere B, Haase D, Jones-Walters L (2016). The science, policy and practice of nature-based solutions: An interdisciplinary perspective. Science of The Total Environment.

[CR44] NWRM. 2015. *Catalogue of natural water retention measures, office International de l’Eau* [online]. Retrieved May 27, 2020, from http://nwrm.eu/measures-catalogue.

[CR45] Opdam P, Bieling C, Plieninger T (2017). How Landscape stewardship emerges out of landscape planning. The science and practice of landscape stewardship.

[CR46] Oteros-Rozas E, Martín-López B, Daw TM, Bohensky EL, Butler JRA, Hill R, Martin-Ortega J, Q
uinlan A (2015). Participatory scenario planning in place-based social-ecological research: Insights and experiences from 23 case studies. Ecology and Society..

[CR48] Pelzer P, Arciniegas G, Geertman S, Lenferink S (2015). planning support systems and task-technology fit: A comparative case study. Applied Spatial Analysis and Policy.

[CR49] Pelzer P, Geertman S, van der Heijden R (2015). Knowledge in communicative planning practice: A different perspective for planning support systems. Environment and Planning B: Planning and Design.

[CR50] Rafiee A, Van der Male P, Dias E, Scholten H (2018). Interactive 3D geodesign tool for multidisciplinary wind turbine planning. Journal of Environmental Management.

[CR51] Raumer, H.S., and Stokman, A. 2013. GeoDesign—Herausforderungen an einen verständigen Umgang mit GIS GeoDesign—A challenge to improve communicating of GIS applications. *In Proceedings of Digital Landscape Architecture*, pp. 311–321.

[CR52] Raymond CM, Frantzeskaki N, Kabisch N, Berry P, Breil M, Nita MR, Geneletti D, Calfapietra C (2017). A framework for assessing and implementing the co-benefits of nature-based solutions in urban areas. Environmental Science and Policy.

[CR53] Reed MS, Challies E, Vente J De, Frewer L, Hohenwallner-Ries D, Huber T, Neumann R, Oughton E (2017). A theory of participation: what makes stakeholder and public engagement 
in environmental management work?. Restoration Ecology.

[CR54] Ryfe DM (2005). does deliberative democracy work?. Annual Review of Political Science.

[CR55] Saathoff W, von Haaren C, Dechow R, Lovett A (2013). Farm-level assessment of CO_2_ and N_2_O emissions in Lower Saxony and comparison of implementation potentials for mitigation measures in Germany and England. Regional Environmental Change.

[CR56] Star SL (2010). This is not a boundary object: Reflections on the origin of a concept. Science Technology and Human Values.

[CR57] Star SL, Griesemer JR (1989). Institutional ecology, `translations’ and boundary objects: Amateurs and Professionals in Berkeley’s Museum of Vertebrate Zoology, 1907–39. Social Studies of Science.

[CR58] Stedman RC (2016). Subjectivity and social-ecological systems: A rigidity trap (and sense of place as a way out). Sustainability Science.

[CR59] Steiner FR, Shearer AW (2016). Geodesign—Changing the world, changing design. Landscape and Urban Planning.

[CR60] Steinitz, C., 2012. *A framework for geodesign: changing geography by design*. Esri.

[CR61] Tashakkori A, Teddlie C, Johnson B, Smelser NJ, Baltes PB (2015). Mixed methods. International encyclopedia of the social & behavioral sciences.

[CR62] Tengö M, Brondizio ES, Elmqvist T, Malmer P, Spierenburg M (2014). Connecting diverse knowledge systems for enhanced ecosystem governance: The multiple evidence base approach. Ambio.

[CR47] van Oudenhoven APE, Schröter M, Drakou EG, Geijzendorffer IR, Jacobs S, van Bodegom PM, Chazee L, Czúcz B (2018). Key criteria for developing ecosystem service indicators to inform decision making. Ecological Indicators.

[CR63] Vonk G, Geertman S (2008). Improving the adoption and use of planning support systems in practice. Journal of Applied Spatial Analysis and Policy.

[CR64] Vonk G, Geertman S, Schot P (2007). A SWOT analysis of planning support systems. Environment and Planning A.

[CR65] Walz U, Stein C (2014). Indicators of hemeroby for the monitoring of landscapes in Germany. Journal for Nature Conservation.

[CR66] Westerink J, Opdam P, van Rooij S, Steingröver E (2017). Landscape services as boundary concept in landscape governance: Building social capital in collaboration and adapting the landscape. Land Use 
Policy.

[CR67] Wissen Hayek U, von Wirth T, Neuenschwander N, Grêt-Regamey A (2016). Organizing and facilitating Geodesign processes: Integrating tools into collaborative design processes for urban transformation. Landscape and Urban Planning.

