# Correction to: Using Geodesign as a boundary management process for planning nature-based solutions in river landscapes

**DOI:** 10.1007/s13280-021-01592-0

**Published:** 2021-06-21

**Authors:** Sarah Gottwald, Jana Brenner, Ron Janssen, Christian Albert

**Affiliations:** 1 Institute for Environmental Planning, Leibniz University Hannover, Hannover, Germany; 2 Department of Spatial Economics, Vrije Universiteit of Amsterdam, Amsterdam, The Netherlands; 3 Institute for Geography, Ruhr-University, Bochum, Germany; 4 Department of Geography, University of Hildesheim, Hildesheim, Germany; 5 Institute of Geography, Chair for Environmental Analysis and Planning in Metropolitan Regions, Ruhr University Bochum, Bochum, Germany

## Correction to: Ambio 10.1007/s13280-020-01435-4

In the original publication, the given name and surname of the author Blal Adem Esmail was swapped in one of the references and hence incorrectly cited as Blal and Geneletti (2017) in the article.

The correct reference is provided below.

Adem Esmail, B., and D. Geneletti. 2017. Design and impact assessment of watershed investments: An approach based on ecosystem services and boundary work. *Environmental Impact Assessment Review* 62: 1–13.

The original article has been corrected.

