# Supplementary material for: Using Geodesign as a boundary management process for planning nature-based solutions in river landscapes
Source: Ambio. 2020 Dec 17;50(8):1477–96. doi: 10.1007/s13280-020-01435-4 (PMC8249630; doi:10.1007/s13280-020-01435-4)
Supplement: Supplementary file 1 — Electronic supplementary material 1 (PDF 717 kb) [file 13280_2020_1435_MOESM1_ESM.pdf]

Ambio

Electronic Supplementary Material

This supplementary material has not been peer reviewed.

Title: Using Geodesign as a boundary management process for planning nature-based solutions in river landscapes

Content

S 1 Workshop participants and institutions 2
S 2 Participants during the Geodesign workshop 3
S 3 Scenarios, derived tasks, and results of the tasks by group 3
S 4 Matrix for climate regulation 4
S 5 Value map climate regulation 4
S 6 Matrix pollination potential 5
S 7 Value map pollinating potential 5
S 8 Perceived nature based recreation 6
S 9 Matrix for nature based recreation 6
S 10 Value map nature based recreation 6
S 11 Matrix for food provision 7
S 12 Value map food provision 7
S 13 Written evaluation by the participants 9

S 1 Workshop participants and institutions, Note:* GDWS is not an official member of the Lila project, but associated as an institution between the WSA and ministries

**Institution (short)**	**Institution (full name)**	**Number of representatives within Lila project**	**Participants in the Geodesign workshop**
SGD Nord	Directorate for Infrastructure and Approval North	1	1
HMUKLV	Hessian Ministry for the Environment, Climate Protection, Agriculture, and Consumer Protection	3	3
GDWS*	General direction Waterway and Shipping	(2)	1
WSA	Waterways and Shipping Office Koblenz	2	2
BfG	German Federal Institute of Hydrology	3	2
RP Gießen	Governmental Authority of Gießen	2	1
MUEEF	Ministry of Environment, Agriculture, Nutrition, Viniculture, and Forestry of Rhineland-Palatinate	1	1
**Total**		**12/(14)**	**11**

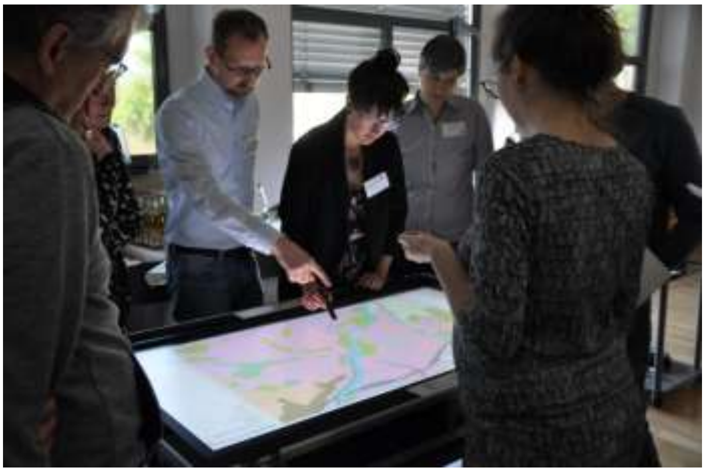

S 2 Participants during the Geodesign workshop, interacting with an Iiyama 55' touch screen (https://iiyama.com/gb_en/products/prolite-t5561uhsc-b1/), ArcGIS 10.6 software and a CommunityViz extension (https://communityviz.city-explained.com/communityviz/index.html). Stakeholders at each touch table were assisted by a facilitator and a GIS support person. An observant took notes of the process.

S 3 Scenarios, derived tasks, and results of the tasks by group

Decision area	'Market' scenario story details	'Market' scenario – derived tasks	'State' scenario story details	'State' scenario – derived tasks
Agriculture	Mix of extensive and intensive	A minimum of 30% of the agricultural areas within the floodplain should be in extensive use	In wetland areas, temporary flooding	Increase the amount of extensive agriculture (cropland and grassland) to 40%
Locks etc.	Without state no power to remove, impacts and uses very complex		If locks stay, ecological connectivity must be assured	
Traffic river	New concept with non-state finance models	Please identify locations for camp grounds, canoe stations, swimming areas, etc. and locate them on the map	For non-motorized boats, emission-free boats with low depth	
Ecological connectivity	No public financing, not economically feasible, too expensive for NGOs		Low for sediment, high for fish	Identify and locate on the map measures for ecological connectivity, for example fish ladders<br>Increase indicator values for climate protection and pollination in at least one river segment
Tech infrastructure in wetlands	Interest conflicts, mainly maintained		Train stays, no new sewage systems in wetlands, bike routes moved away from river	Identify and locate on the map recreational opportunities, such as cycling paths, beaches, etc.
Settlement	No extension within floodplains in rural areas, urban areas grow (due to citizen engagement)	Increase built-up environment by 20ha without reducing values of the indicator pollination and climate protection	No more construction in wetland areas, partly removal	Reduce the area of built-up environment in the morphological floodplain from 320 ha to 250 ha
Protected areas	No state regulation, private actors cannot do this task	Please locate on the map areas of especial importance for agriculture, recreation, nature protection	Increase where possible	Please identify on the map areas of especial importance for agricultural use (with temporal flooding), for the expansion of the floodplain, recreation and nature protection
Recreation/Tourism	Many small opportunities	Increase recreational value in at least one land use parcel (from red to orange, or from orange to green)	Maybe regulation with vignettes	Sustain the existing recreational quality despite changes in land use

		Please identify potential bike and hiking trails and locate them on the map		
Services and industry	Number of services	Allocate 10 ha of industrial area.	Tourism services – increase, industry – decrease	Reduce industrial area within the floodplain from 290ha to 250ha
Health/well-being	Heterogeneous and individual, high quantity, low standards by companies		Access to the river for everybody	Allocate paths and entry points to enable access to the river
Knowledge and education	More personal responsibility		Learning with and in nature, sustainability education	
River			Restoration	
Wetland	Reactivate small wetland forest areas	Allocate 40 ha of forest on the floodplain		

S 4 Matrix for climate regulation

Land use	soil type	
	**Fluvisol, Pseudogley, Gley or Podsol**	**others**
Cropland of intensive use	low*	low
Cropland of extensive use	low*	low
Permanent crops	medium	low
Urban green spaces	medium	low
Grassland of intensive use	medium	low
Grassland of extensive use	medium	low
Industry	low	low
Deciduous forest	high	medium
Coniferous and mixed forest	high	medium
Lakes and ponds	low	low
Settlement	low	low
Woody and marginal vegetation	medium	low
Traffic infrastructure	low	low

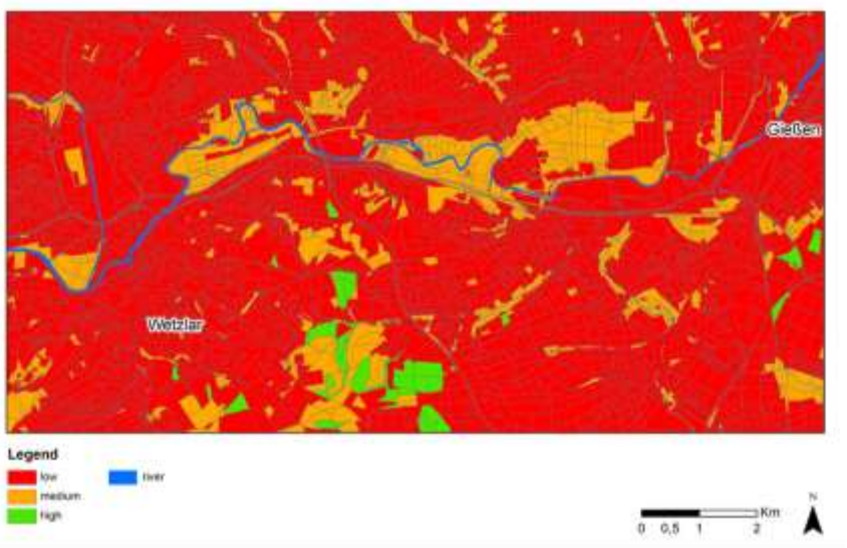

S 5 Value map climate regulation

S 6 Matrix pollination potential

Land use	pollination
Cropland of intensive use	medium
Cropland of extensive use	medium
Permanent crops	medium
Urban green spaces	medium
Grassland of intensive use	high
Grassland of extensive use	high
Industry	low
Deciduous forest	medium
Coniferous and mixed forest	medium
Lakes and ponds	low
Settlement	low
Woody and marginal vegetation	high
Traffic infrastructure	medium

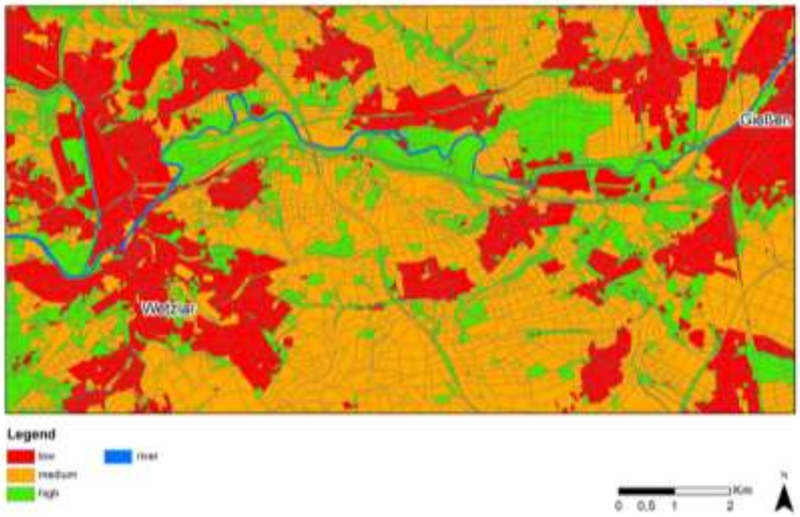

S 7 Value map pollinating potential

S 8 Perceived nature based recreation (Hermes et al. 2018)

Land use	Nature-based recreation (perceived naturalness)
Cropland of intensive use	medium
Cropland of extensive use	medium
Permanent crops	medium
Urban green spaces	medium
Grassland of intensive use	medium
Grassland of extensive use	high
Industry	low
Deciduous forest	high
Coniferous and mixed forest	low
Lakes and ponds	high
Settlement	low
Woody and marginal vegetation	high
Traffic infrastructure	low

S 9 Matrix for nature-based recreation

Current user frequency	Nature-based recreation (perceived naturalness)		
	**high**	**medium**	**low**
**high**	high	high	medium
**medium**	high	medium	medium
**low**	medium	medium	low

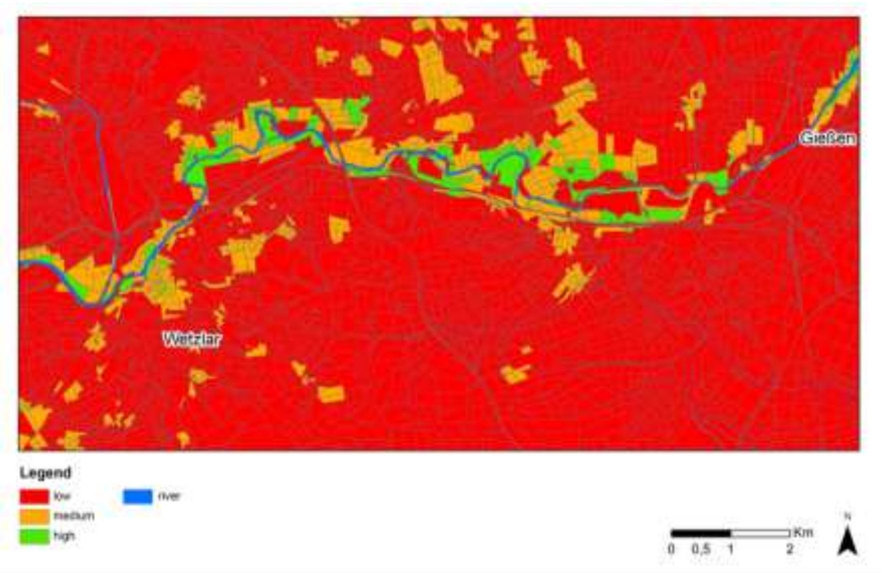

S 10 Value map nature based recreation

S 11 Matrix for food provision

Land use	Food provision
Cropland of intensive use	high
Cropland of extensive use	medium
Permanent crops	high
Urban green spaces	low
Grassland of intensive use	high
Grassland of extensive use	medium
Industry	low
Deciduous forest	low
Coniferous and mixed forest	low
Lakes and ponds	low
Settlement	low
Woody and marginal vegetation	low
Traffic infrastructure	low

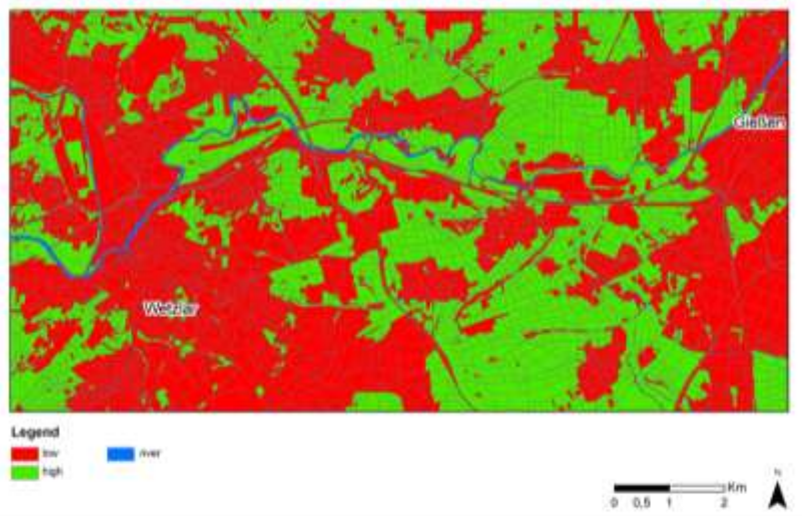

S 12 Value map food provision

S 13 Box 2 representing detailed results

Box 2 Detailed results

Sketch maps of the Market scenario

The Market scenario sketch maps developed by both stakeholder groups differ substantially. While Group A, at the large touch table, identified seven priority areas with a total size of 813 ha, participants in Group B identified ten areas of importance of about 1200 ha. Group A's map shows drawings between river segment 3 and 14, clustered in the lake region (segments 11 to 14) and in proximity to the major city in this study region (segments 5 to 8, Fig.6). In both clusters, areas for nature (a total of 347 ha), agriculture (267 ha) and recreation (199 ha) have been highlighted. Inside the city there is a large nature priority area. In contrast, participants in Group B chose areas of importance along a wider stretch (segments 1 to 15), and proposed a greater share of area for

recreation (508 ha). Priority areas drawn by Group B are more equally distributed than those of Group A, but also cluster around the lake region. In contrast to Group A's sketch, the city centre of Wetzlar shows a priority for recreation. In Group A's scenario sketch map the identified points of interest are spread out between the third and the last (17th) river segment, and these are designated for canoeing, camping and swimming (amounting to one area each). A cycling path and hiking trail follow the Lahn through the whole study area. Group B likewise introduced a cycling and hiking path, but it only covers half the distance, extending from the east of the study area to Dorlar. The points of interest are spread along the river, and there is one for camping and two for canoeing.

Land use change maps and impact assessments in the Market scenario

The Market scenario map of Group A shows 126 changed parcels and a total of 360 ha. Most changes relate to grassland: 255 ha (almost 40% of grassland within the morphological floodplain) was converted, mostly into extensive grassland (52%) and woody and marginal vegetation (20%). The second most frequently changed land use was intensive cropland: 66 ha were changed (16.4% of all cropland), of which 93% was turned into extensive cropland. The most frequently allocated land use was extensive grassland (147 ha), followed by deciduous forest (79 ha) and extensive cropland (61 ha, Fig. 6). This shows impacts on food provision, which decreased in almost all river segments (Tab. 2, Fig.6). In the lake region extensive agriculture, forest and woody and marginal vegetation was predominantly allocated. These had a positive impact on the climate regulation value, which in the 12th segment increased from 1.59 (low) to 1.7 (medium). In urban areas, industry as well as grassland was converted to settlement or industrial uses (Fig.6). The impacts on pollination values can be observed in segment 15, where these decreased from 1.73 (medium) to 1.59 (low). Changes in recreation values were minor in terms of actual numbers, yet they crossed the set threshold and, thus, became visible during the workshop's second segment, showing a change from medium to low.

The spatial translation of the same scenario differs slightly for Group B, where changes affected more than twice the area (639 ha). Similarly to the previously described map (Group A), intensive grassland lost most area (360 ha, which is 55% of the grassland in the morphological floodplain), followed by cropland (194 ha, or 48.5% of the cropland inside the floodplain). This negatively impacted food production. Again, many changes were made in the lake region, but these extend also along the river in segments 9, 10 and 11 (Fig.6). Most of the intensive grassland was converted into extensive grassland (73%). Intensive agriculture was converted to urban green (30%), industry (22%) and extensive grassland (20%). The most frequently allocated land use was extensive grassland (314 ha), followed by urban green (89 ha) and deciduous forest (71 ha, Fig. 5). The latter had negative impact on pollination, which can be observed, for example, in segment 2, where the value decreased from 1.81 (medium) to 1.6 (low). At the same time this had a positive impact on climate regulation values, which in the same segment increased from 1.18 (low) to 1.77 (medium, Tab.2, Fig.6).

[Tab.2]
[Fig.6]

Sketch maps of the State scenario

The translation of the scenario narratives into spatial scenarios differed between the groups. In the scenario map developed by Group A, four important areas (506 ha) were allocated, one for each category (184 ha nature, 115 ha recreation, 113 ha agriculture, 94 ha new floodplain; Fig.7). Group B allocated more priority areas (six), but a smaller area (460 ha). The two nature priority areas represent 256 ha – considerably more than Group B had allocated for this same scenario. The extent of agriculture is similar (116 ha), while that of recreation is much smaller (79 ha). Priority areas allocated by Group A are exclusively located in the lake region. In contrast, Group B prioritised areas between the 7th and 15th river segments. The long stretch of nature priority area on the northern lakes and along the river was termed 'blue ribbon', with the idea of enhancing ecological connectivity. The map produced by Group A shows 19 points of interest spread between the 3rd and

17th river segments; these are concentrated in the urban areas and one river meander and include 11 fish ladders, 7 entry points for canoes, and one beach. The cycling path/hiking trail also stretches through the whole study area. In contrast, Group B located a biking trail along around one third of the river's length, and there are only two points of interest dedicated to river accessibility.

Land use change and impact assessment in the State scenario
The State scenario map shows 205 changed parcels (714 ha), mostly for intensive grassland, where 412 ha (67% of the total cropland within the morphological floodplain) have been changed, mainly into extensive grassland (40%) and deciduous forest (26%). Further, 49% of the intensive cropland within the morphological floodplain were changed (196 ha), mainly into extensive grassland (40%) and extensive cropland (40%). This had a negative impact on food provision, which decreased in almost all segments. Also, pollination decreased when grassland was converted to forest, e.g. in segment 12, where this decreased from 2.47 to 2.22. At the same time, this had a positive impact on recreation, which increased from 2.3 (medium) to 2.45 (high). The most frequently allocated land uses were extensive grassland (272 ha), followed by deciduous forest (182 ha) and woody and marginal vegetation (109 ha, Fig.6). Around the lake region and river segments 5 and 6 forests, permanent crops, and woody and marginal vegetation are new land uses (Fig.7). This had positive impacts on carbon regulation, which for example increased in segment 6 from 1.44 (low) to 1.77 (medium).

The map co-produced by Group B shows 168 changed parcels with a total area of 455 ha, mostly affecting intensive cropland (245 ha and 61% of the entire morphological floodplain) with conversions mainly into extensive cropland (58%) and intensive grassland (25%). The most frequently allocated land use was extensive cropland (151 ha), followed by extensive grassland (145 ha) and woody and marginal vegetation (89 ha, Fig.5, Tab.3). Again, this had a considerable negative impact on food provision values. The 11th and 15th segments show large changes, with new areas for forest and urban green (Fig.7) leading to an increase of climate regulation indicator values from 1.46 (low) to 1.84 (medium) and pollination (from 2.25 (medium) to 2.32 (high)) in segment 11. There were minor increases of recreation values due to extensification of cropland, e.g. in segment 9.

[Tab.3]
[Fig.7]

S 14 Written evaluation by the participants

Positive	Negative	Open Q
1. How the touchtable was used<br>2. Good approach for visualization<br>3. Good insight into the "tool" touchtalbe<br>4. Working together at the touch table<br>5. Innovative approach, good organisation, helpful exchange with lila partners<br>6. New possibility/ technique for citizen participation	1. Comparison of the scenarios<br>2. Relation of the "meaningful places" to scenarios not obvious<br>3. To many river segments, therefore confusing and too much<br>4. System crashes cost time, limitation to fewer river segments would be better, impact of indicators not very clear	1. What could be done to change indicators<br>2. More categories<br>3. "evaluation tool" limited to 4 factors, which do not sufficiently reflect the complexity of ecosystem services<br>4. Tasks for group work should be less extensive<br>5. Data basis and calculations must be adapted to the purpose (participants,

7. Got to know well usable new method, appropriate to start conversation, very graphic<br>8. Interesting tool (touchtable), interesting discussion within group<br>9. Appropriate communication tool; talk, “draw”, evaluate quickly in one process<br>10. Great workshop place, good workshop preparation, good working atmosphere	5. Comparison of maps difficult<br>6. Time short (tasks too large)<br>7. One scenario too much, simpler graphics, no water related measures, how high was the [preparation] effort?<br>8. Time management, lunch break too short, too little time for exchange with participants<br>9. Time was too short	tasks) → a lot preparation upfront<br>6. Disclose the algorithm for the evaluation, define new areas more accurately, simplify graphic
